# Cross-sectional and longitudinal associations of Iron biomarkers and cardiovascular risk factors in pre- and postmenopausal women: leveraging repeated measurements to address natural variability

**DOI:** 10.1186/s12933-024-02242-x

**Published:** 2024-05-07

**Authors:** Noushin Sadat Ahanchi, Amira Salomé Fischer, Hugo G. Quezada-Pinedo, Farnaz khatami, Michele F. Eisenga, Taulant Muka, Pedro-Marques Vidal

**Affiliations:** 1 Institute of Social and Preventive Medicine, University of Bern, Bern, Switzerland; 2 Graduate School for Health Sciences, University of Bern, Bern, Switzerland; 3 Department of Cardiology, Bern University Hospital, University of Bern, Bern, Switzerland; 4 The Generation R Study Group, Rotterdam, Netherlands; 5 Division of Nephrology, Department of Internal Medicine, University of Groningen, Groningen, Netherlands; 6 Community Medicine Department, Tehran University of Medical Sciences, Tehran, Iran; 7 Department of Internal Medicine, Internal Medicine, Lausanne University Hospital, Lausanne, Switzerland; 8 Epistudia, Bern, Switzerland

**Keywords:** Iron biomarkers, Menopause, Cohort, Cardio-metabolic risk factors

## Abstract

**Background:**

The association between iron biomarkers and cardiovascular disease risk factors (CVD-RFs) remains unclear. We aimed to (1) evaluate the cross-sectional and longitudinal associations between iron biomarkers (serum ferritin, transferrin saturation (TSAT), transferrin) and CVD-RFs among women, and (2) explore if these associations were modified by menopausal status.

**Method:**

Cross-sectional and longitudinal analyses including 2542 and 1482 women from CoLaus cohort, respectively. Multiple linear regression and multilevel mixed models were used to analyse the associations between Iron biomarkers and CVD-RFs. Variability of outcomes and iron markers between surveys was accessed using intraclass correlation (ICC).

**Results:**

After multivariable adjustment, elevated serum ferritin levels were associated with increased insulin and glucose levels, while higher transferrin levels were linked to elevated glucose, insulin and total cholesterol, and systolic and diastolic blood pressure (p < 0.05). No association was observed between CVD-RFs and TSAT (p > 0.05). Iron biomarkers demonstrated low reliability across reproductive stages but exhibited stronger associations in the perimenopausal group. In longitudinal analysis, we found association only for transferrin with lower glucose levels [β = − 0.59, 95% CI (− 1.10, − 0.08), p = 0.02] and lower diastolic blood pressure [β = − 7.81, 95% CI (− 15.9, − 0.56), p = 0.04].

**Conclusion:**

In cross-sectional analysis, transferrin was associated with several CVD-RFs, and the associations did not change according to menopausal status. Conversely, in the longitudinal analyses, changes in transferrin were associated only with lower glucose and diastolic blood pressure levels. These differences might stem from the substantial longitudinal variation of iron biomarkers, underscoring the need for multiple iron measurements in longitudinal analyses.

**Supplementary Information:**

The online version contains supplementary material available at 10.1186/s12933-024-02242-x.

## Key Messages

- Different iron biomarkers showed inconsistent associations with cardiovascular disease risk factors (CVD-RFs) in cross-sectional and longitudinal analyses.
- We assessed the cross-sectional and prospective associations between several iron biomarkers and CVD-RFs in women, and the possible role of menopause.
- In cross-sectional analyses, transferrin was associated with several CVD-RFs in women, which was not the case for ferritin or transferrin saturation.
- In the longitudinal part of our study, we took advantage of repeated measures of iron biomarkers to correct for low reliability of iron biomarkers.
- In the longitudinal analyses, we found that only changes in transferrin were associated with lower levels of glucose and diastolic blood pressure.
- The associations between iron biomarkers and CVD-RFs did not change according to menopausal status.

**Supplementary Information:**

The online version contains supplementary material available at 10.1186/s12933-024-02242-x.

## Introduction

Regardless of the geographical variations, cardiovascular disease (CVD) remains the most common cause of death worldwide [1]. Sex differences in CVD are well-documented, encompassing variations in risk factors, disease presentation, and outcomes between men and women [2]. While men traditionally exhibit higher rates of CVD, women often catch up in risk post menopause, suggesting hormonal influences on cardiovascular health [3].

Menopause, characterized by hormonal shifts and decreased estrogen levels, represents a pivotal sex-specific event that significantly impacts CVD risk profiles and outcomes in women [4]. While oestrogen deficiency has been suggested as the main causative factor for the increased risk of cardiometabolic diseases after menopause, the evidence is not persuasive in supporting this hypothesis and is amenable to alternative explanations [5].

The associations between iron biomarkers and CVD-RFs did not change according to menopausal status.

During menopause, oestrogen levels decrease by 90%, while risk of iron deficiency decreases because of decreasing menstrual periods [6, 7]. Thus, changes in iron status have been suggested as an alternative explanation for the increased risk of cardiometabolic disease after menopause [8]. Previous studies have linked iron status with the risk of heart failure, CVD, and diabetes mellitus [9–11]. A possible mechanism might be the role of iron metabolism on cardiovascular risk factors, including blood lipids, blood pressure, and glucose levels [11]. Indeed, prior cross-sectional studies have shown iron to be negatively associated with HDL cholesterol and positively associated with fasting blood sugar, triglyceride and blood pressure levels [11]. However, the results are inconsistent [12]. This inconsistency could be due to several factors such as (1) small sample size; (2) differences in sampling methods; (3) non-reliable measures of iron status; (4) results based on a single measurement of iron status (which can fluctuate markedly within individuals over time); (5) presence of potential confounding variables; (6) ethnic differences in iron biomarkers, and (7) lack of replication using a unified methodology [13]. Further, longitudinal studies with repeated measurements of iron biomarkers are lacking [11].

Understanding the intricate interplay between iron biomarkers and cardiovascular risk factors in pre- and postmenopausal women holds significant promise in elucidating underlying biological pathways that may contribute to CVD development. Moreover, investigating these biomarkers could offer valuable insights into their potential clinical utility in risk prediction, prevention, and management strategies [13]. Hence, this study aimed to assess the association between iron biomarkers and cardiovascular disease risk factors (CVD-RFs–cholesterol, blood pressure, glucose metabolism) in two ways. Firstly, we explored the interaction between menopause and iron status regarding the association between iron status and CVD-RFs in the total sample. Secondly, we evaluated the cross-sectional and longitudinal associations between iron status and CVD-RFs among pre- and postmenopausal women. In addition, we explored the intra-variability of iron biomarkers over time as it determines whether a single measurement accurately reflects an individual's iron status. This information is critical for guiding clinical decision-making and interventions aimed at reducing CVD risk by ensuring the accuracy of iron biomarker assessments.

## Methods

### Participants

The CoLaus study is an ongoing population-based cohort conducted in the city of Lausanne, Switzerland, aiming to identify the biological and genetic determinants of cardiovascular disease [14]. Briefly, all participants aged 35 to 75 living in the city of Lausanne were eligible. Subjects were included if they consented to participate in the study and were willing to provide a blood sample. Recruitment began in June 2003 and ended in May 2006. The first follow-up was performed between April 2009 and September 2012, and the second follow-up between May 2014 and April 2017. The information collected at follow-ups was the same as that collected during the baseline examination. For this study, data from the baseline, first, and second follow-ups of the CoLaus study were used.

### Selection of participants

**Fig. 1:**
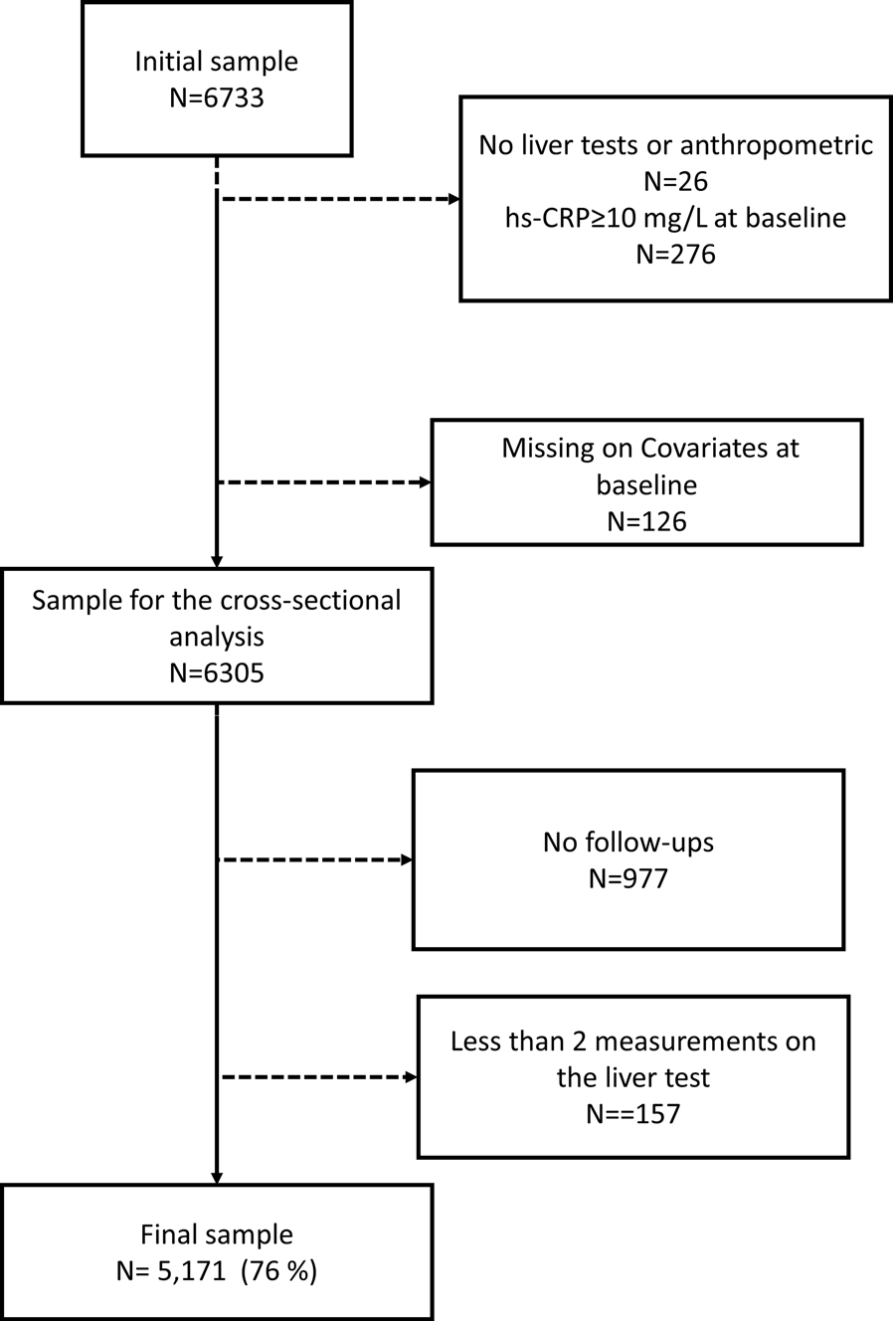
Enrolment flow chart for study population

Overall, 3544 women aged 35–75 years from the baseline survey were eligible. Of these, 868 (24.4%) were excluded due to missing information on CVD-RFs, and 134 (3.8%) because of C-reactive protein (CRP) levels ≥ 10 mg/L, as they are indicative of acute inflammatory processes that might modify levels of iron markers. This led to 2542 women (71.7%) for the cross-sectional analysis (Fig. 1). Among the 2542 women, 433 (17.0%) were excluded because they had less than two measurements on CVD-RFs, 314 (12.3%) because of missing iron biomarkers, and 268 (10.5%) because of CRP levels ≥ 10 mg/L during follow-up. A further 49 women (2.0%) were excluded because of missing or unreliable information on menopausal status (i.e., reporting being postmenopausal at the first follow-up and premenopausal at the second follow-up). Thus, 1482 women (58.3% of the participants in the cross-sectional analysis) were included in the longitudinal analysis (Fig. 1).

### Iron status

A comprehensive assessment of iron status often involves considering multiple biomarkers. Ferritin is a long-term indicator of iron stores in the body; elevated levels indicate high iron stores, whereas low levels may indicate iron deficiency. Serum iron levels provide a snapshot of the amount of iron circulating in the blood. Interpretation of serum iron levels in isolation can be challenging due to their daily variability [15]. Transferrin plays a key role in transporting iron throughout the body and is often measured indirectly through calculations involving other iron biomarkers; abnormal levels may indicate disruptions in iron metabolism or transport. Transferrin saturation (TSAT) is the ratio of serum iron to transferrin, is a more dynamic marker of short-term iron status compared to ferritin. Elevated TSAT levels may suggest excess iron accumulation or impaired iron regulation, while low TSAT levels may indicate iron deficiency or inefficient iron absorption [16]. Venous blood samples were drawn after an overnight fast. Clinical chemistry assays were performed at the central laboratory of the University Hospital of Lausanne (CHUV). Iron was assessed by colorimetric method (ferrozine, BioSystems); ferritin (µg/L) by immunoturbidimetric method (Tina-quant 4th generation, Roche Diagnostics, Switzerland); transferrin (µg/dL) by immunoassay. Transferrin saturation (TSAT, %) was determined as (serum iron ÷ (25 × transferrin)) × 100 [17].

### Cardiovascular risk factors

Blood pressure and heart rate were measured thrice on the left arm, with an appropriately sized cuff, after at least 10-min rest in the seated position using an Omron® HEM-907 automated oscillometric sphygmomanometer (Matsusaka, Japan). The average of the last two measurements was used for analyses. Body weight and height were measured in light indoor clothes and without shoes. Body weight was measured in kilograms to the nearest 100 g using a Seca® scale, which was calibrated regularly. Height was measured to the nearest 5 mm using a Seca® height gauge. Body Mass Index (BMI) was calculated as weight (kg) divided by the square of the height (m). Serum lipids and glucose were measured using enzymatic colorimetric assays. Insulin was assessed by electrochemiluminescence (ECLIA). High-sensitivity CRP was assessed by immunoassay [14].

### Menopausal status

Women participating in the study were asked whether they were still having menses. Women reporting “no” were classified as postmenopausal and as premenopausal if they answered “yes.” Based on the self-reported menopausal status at the first and second follow-up visits, women were classified as being (1) premenopausal-premenopausal if they remained premenopausal, (2) premenopausal-postmenopausal if they changed their status, (3) and otherwise as postmenopausal.

### Covariates

Information on age, lifestyle, educational attainment, medical history (CVD, diabetes), medication use (antihypertensive drugs, antidiabetic drugs or statins) and alcohol consumption was obtained through a questionnaire. Educational attainment was defined as ‘high’ for those with at least a university degree, ‘middle’ for those who finished secondary school and ‘low’ for those who completed mandatory elementary education or apprenticeship. Alcohol consumption was obtained by asking if participants regularly consumed alcohol and their weekly consumption of wine, beer, and spirits in units per week. Smoking was categorized as never, former, and current. Body mass index and C-Reactive Protein (CRP) were considered as continuous variables. Data on hormone replacement therapy (HRT) was obtained and categorized as having ever taken HRT (‘yes’) or not (‘no’).

### Statistical analysis

Statistical analyses were performed using Stata version 15.1 for Windows (Stata Corp, College Station, TX, USA). Baseline characteristics of the study population are described as frequencies and percentages for categorical variables, mean and standard deviation, or median and 25th–75th percentile for continuous variables. The Gaussian distribution of continuous variables was visually inspected using a histogram and applying the Shapiro–Wilk test. Skewed variables (iron biomarkers, insulin, and hs-CRP) were log-transformed to achieve a normal distribution. Iron markers were corrected for CRP as suggested by others [18, 19] prior to analysis. Two sets of analyses were performed: cross-sectional and longitudinal.

### Cross-sectional analysis

For the cross-sectional analysis baseline iron data, confounders and CVD-RFs were used. We compared the sociodemographic, iron biomarkers, CVD-RFs, and other lifestyle variables between pre- and postmenopausal women, using the independent-samples T-test or Mann–Whitney-U test for continuous variables, and the chi-squared test for categorical variables. Multiple linear regression models were used to investigate the association of CVD-RFs (as dependent variables) with levels of iron biomarkers (ferritin, transferrin, and TSAT, as independent variables). Three models were used; the first one included age only, while the second one additionally included BMI, smoking, alcohol use, educational levels, hormone replacement therapy (HRT), antidiabetic and antihypertensive drugs, CVD, and menopause status, when the outcome was insulin or glucose, diabetes was also included in the model. The third one stratified by menopause status and included age BMI, smoking, alcohol use, educational levels, hormone replacement therapy (HRT), antidiabetic and antihypertensive drugs, CVD, When the outcome was insulin or glucose, diabetes was also included in the model. All iron biomarkers were corrected for CRP as suggested by BRINDA protocol prior to analysis. Results were expressed as beta coefficient and 95% confidence interval.

### Variability of outcomes and iron markers

Variability of outcomes and iron markers between surveys was accessed using intraclass correlation coefficient (ICC). ICC was obtained from a two-way random effects model with measures of absolute agreement. An ICC ≥ 0.75 was considered excellent, between 0.40 and 0.75 good and < 0.40 unsatisfactory [20]. To explore the role of menopausal status, we calculated ICC in the whole sample and according to menopausal status (pre-pre, pre-post and post-post).

### Longitudinal analysis

To investigate the longitudinal association between iron biomarkers and CVD-RFs, we used a multilevel mixed-model approach for baseline, first, and second follow ups, including the same baseline confounders as in the cross-sectional analysis. Our model incorporated both fixed and random effects to comprehensively account for individual variability and potential confounding factors. The fixed effects included iron biomarkers, follow-up time, and their interaction term, together with the same baseline confounders as in the cross-sectional analysis. The fixed effects elucidated how changes in iron biomarkers over time were associated with changes in CVD-RFs. The random effects comprised random intercepts and random slopes, capturing individual-level variability in baseline CVD-RF levels and their rates of change over time.”. For missing values during follow-up (smoking and HRT, up to 29% missing data), multiple imputations were conducted using a predictive mean matching method and 5-time imputation with 50 iterations using Stata.

### Sensitivity analyses

We performed three different sensitivity analyses. Firstly, we added an interaction term between iron biomarkers and menopausal status using the whole sample to explore the role of menopause on the associations between iron markers and CVD-RFs. Secondly, we stratified all analyses by menopausal status. Thirdly, a sensitivity analysis was conducted to further explore the association between the iron biomarkers and CVD-RFs. In this analysis, we categorized the iron biomarker variable into tertiles based on their relative levels and tested for a linear trend in cross-sectional and longitudinal analyses. This approach allowed us to assess potential non-linear relationships and investigate whether there were different patterns of association across different ranges of the iron biomarkers.

## Results

### Cross-sectional analysis

The main characteristics of the women included according to menopausal status are summarized in Table 1. As compared to premenopausal women, postmenopausal women had higher ferritin, BMI, CRP, and SBP levels, more prevalent CVD and diabetes, reported higher consumption of anti-diabetic and antihypertensive medications, and had lower transferrin levels. Also, the comparison between included and excluded participants showed differences in age (p < 0.001), BMI (p = 0.03), use of antidiabetic drugs (p = 0.02), and alcohol consumption (p = 0.01). Included participants exhibited higher mean age, BMI, a greater percentage reported antidiabetic drug use, and a higher prevalence of alcohol consumption compared to excluded participants (Additional file 1: Table S1).

**Table 1:** Characteristic of the study participants at baseline, CoLaus study, Lausanne, Switzerland

Variable	Total	Menopause	Pre-menopause	P value*
Sample size	2542	1421	1121	
Age (years)	53.2 (10.4)	60.8 (6.1)	44.1 (5.3)	< 0.001
Smoking status (%)				0.53
Never	1219 (47.9)	726 (51.09)	493 (43.9)	
Former	718 (28.2)	407 (28.6.7)	311 (27.7)	
Current	605 (23.8)	288 (20.2)	317 (28.2)	
Education level (%)				0.37
High	379 (14.9)	140 (9.8)	239 (21.3)	
Middle	633 (24.9)	328 (23.1)	305 (27.2)	
Low	1530 (60.1)	951 (63.3)	577 (51.4)	
Use of antihypertensive drugs (n, %)	404 (15.8)	335 (23.5)	69 (6.1)	0.02
Use of antidiabetic drugs (n, %)	53 (2.1)	49 (3.4)	4 (0.3)	0.03
Alcohol drinker	1,611 (63.3)	894 (62.9)	717 (63.1)	0.06
Body mass index (kg/m2)	25.2 (4.6)	26.00 (4.2)	24.2 (4.3)	0.04
High-sensitivity C-reactive protein, (mg/l)	1.3 (0.6–2.7)	1.6 (0.8–2.9)	1 (0.5–2.4)	0.06
Prevalence of CVD	125 (4.9)	105 (7.3)	20 (1.7)	0.03
Prevalence of diabetes	94 (3.7)	82 (5.7)	12 (1.1)	0.04
HRT user (%)	905 (35.6)	850 (59.8)	55 (0.49)	0.001
Cardiovascular risk factors				
Glucose (mmol/L)	5.30 (0.7)	5.43 (0.8)	5.15 (0.4)	0.05
Insulin (mIU/mL)	6.01 (4.4–9.5)	6.57 (4.6–9.8)	5.9 (4.8–8.4)	0.44
SBP (mm Hg)	124 (17)	130 (18)	116 (13)	0.04
DBP (mm Hg)	77 (11)	79 (10)	75 (10)	0.82
HDL-C (mmol/L)	1.80 (0.4)	1.83 (0.43)	1.87 (0.4)	0.60
TC (mmol/L)	5.62 (1.1)	5.98 (0.98)	5.19 (0.9)	0.28
LDL (mmol/L)	3.30 (0.93)	3.57 (0.91)	3.21(0.88)	0.07
Triglyceride (mmol/L)	1 (0.8–1.4)	1.1 (0.9–1.6)	0.9 (0.7–1.2)	0.11
Iron biomarkers				
Iron (μg/dL)	95 (74–119)	96 (77–122)	97 (77–118)	0.05
Ferritin ( μg/L)	78 (44–138)	112 (66–169)	52 (30–86)	0.002
Transferrin (mg/dL)	236 (213–263)	232 (211–256)	239 (215–271)	0.04
Transferrin saturation (%)	29.5 (22.4–36.6)	30.1 (23.4–36.4)	28.5 (20.7–37.3)	0.06

Continuous variables shown as mean (SD) with *p* according to *t*-test; categorical variables as % with *p* according to *χ*^2^, median (25th-75th percentile) with *p* according to Mann–Whitney *U*-test

CVD-RFs: Cardiovascular disease risk factors, CVD: Cardiovascular disease, HDL-C: High density lipoprotein cholesterol, LDL: Low-Density Lipoprotein, BMI, body mass index. SBP, systolic blood pressure; DBP, diastolic blood pressure; TC: Total cholesterol, HRT: Hormone Replacement Therapy

^*^Comparing menopause and Pre-menopause women respondents

The results of the multivariable analysis of the associations between CVD-RFs and iron markers are summarized in Table 2. In the age adjusted model, Log-transferred ferritin and transferrin were positively associated with glucose and insulin (p < 0.05). In addition, Log-transferred transferrin was positively associated with SBP, DBP, TC in model 1 (p < 0.05) (Table 2). After adjustment for potential confounders, ferritin levels were positively associated with insulin and glucose, while transferrin levels were positively associated with glucose, insulin, SBP, DBP and TC (Table 2). No association between CVD-RFs and TSAT was found in the age and in the fully adjusted models (p > 0.05) (Table 2), and no significant quadratic or cubic term was found (p > 0.05) (Additional file 1: Table S3).

**Table 2:** Results of linear regression analysis of the associations between iron biomarkers and cardiovascular risk factors at baseline, CoLaus study, Lausanne, Switzerland

	Total, model 1			Total, model 2			Menopause, model 3		Pre− menopause, model 3	
	Beta (95% CI)	P-value	P-value §	Beta (95% CI)	P-value	P-value §	Beta (95% CI)	P-value	Beta (95% CI)	P-value
Sample size	2542			2542			1421		1121	
Glucose (mmol/L)										
Ferritin	0.05 (0.001; 0.10)	0.01	0.69	0.03 (0.009; 0.07)	0.04	0.55	0.01 (− 0.04; 0.08)	0.61	0.04 (− 0.00; 0.08)	0.06
Transferrin	0.40 (0.16; 0.64)	< 0.001	< 0.001	0.21 (0.02; 0.41)	0.03	0.01	0.48 (0.15; 0.81)	< 0.001	0.02 (− 0.17; 0.22)	0.93
TSAT	− 0.14 (− 0.24; 0.05)	0.07	0.05	− 0.05 (− 0.12; 0.02)	0.17	0.43	− 0.07 (− 0.21; 0.06)	0.40	− 0.05 (− 0.12; 0.01)	0.17
Insulin (mIU/mL)										
Ferritin	0.04 (0.002; 0.07)	0.01	0.38	0.03 (0.001; 0.06)	0.03	0.55	0.08 (0.03; 0.13)	< 0.001	0.04 (0.001; 0.09)	0.03
Transferrin	0.48 (0.31; 0.64)	< 0.001	0.60	0.44 (0.29; 0.59)	< 0.001	0.83	0.40 (0.15; 0.66)	0.02	0.18 (− 0.03; 0.41)	0.09
TSAT	− 0.09 (− 0.16; 0.03)	0.06	0.21	− 0.03 (− 0.09; 0.02)	0.27	0.78	− 0.04 (− 0.15; 0.05)	0.41	− 0.01 (− 0.09; 0.06)	0.38
SBP (mm Hg)										
Ferritin	0.20 (− 0.78; 1.20)	0.68	0.53	0.12 (− 0.87; 1.11)	0.81	0.74	− 0.27 (− 1.90; 1.34)	0.73	0.66 (− 0.52; 1.84)	0.27
Transferrin	9.04 (3.99; 14.10)	< 0.001	0.47	8.80 (3.79; 13.81)	< 0.001	0.57	9.80 (3.79; 14.80)	< 0.001	10.18 (4.37; 16.0)	< 0.001
TSAT	− 2.15 (− 4.09; 0.21)	0.11	0.98	− 1.46 (− 3.39; 0.47)	0.64	0.89	− 1.41 (− 4.85; 2.02)	0.82	− 1.52 (− 3.62; 0.58)	0.44
DBP (mm Hg)										
Ferritin	0.07 (− 0.59; 0.73)	0.83	0.85	− 0.10 (− 0.75; 0.54)	0.76	0.67	− 0.37 (− 1.36; 0.61)	0.45	− 0.03 (− 0.90; 0.84)	0.94
Transferrin	4.81 (1.44; 8.19)	< 0.001	0.10	5.00 (1.71; 8.29)	< 0.001	0.17	3.19 (− 1.81; 8.20)	0.21	7.26 (2.96; 11.56)	0.04
TSAT	− 1.53 (− 2.83; 0.24)	0.13	0.17	− 0.94 (− 2.21; 0.31)	0.14	0.14	− 0.05 (− 2.15; 2.04)	0.85	− 1.70 (− 3.25; 0.11)	0.05
HDL (mmol/L)										
Ferritin	− 0.007 (− 0.03; 0.02)	0.60	0.07	− 0.001 (− 0.02; 0.02)	0.91	0.03	− 0.02 (− 0.06; 0.01)	0.17	0.02 (− 0.01; 0.05)	0.27
Transferrin	0.02 (− 0.11; 0.17)	0.70	0.28	0.04 (− 0.08; 0.18)	0. 49	0.47	0.005(− 0.19; 0.20)	0.95	0.09 (− 0.08; 0.28)	0.29
TSAT	0.11 (− 0.05; 0.16)	0.07	0.29	0.07 (− 0.01; 0.12)	0.12	0.87	0.07 (− 0.00; 0.16)	0.51	0.06 (− 0.00; 0.12)	0.09
TC (mmol/L)										
Ferritin	0.01 (− 0.04; 0.07)	0.63	0.84	0.001 (− 0.05; 0.07)	0.76	0.32	− 0.001 (− 0.10; 0.09)	0.90	− 0.11 (− 0.08; 0.07)	0.86
Transferrin	0.09 (0.002; 0.14)	0.02	0.05	0.03 (− 0.004; 0.19)	0.04	0.01	0.35 (− 0.84; 0.40)	0.16	0.30 (0.13; 0.47)	< 0.001
TSAT	0.02 (− 0.09; 0.15)	0.76	0.52	0.002 (− 0.11; 0.12)	0.67	0.83	− 0.00 (− 2.20; 0.20)	0.74	− 0.01 (− 0.14; 0.13)	0.07
LDL (mmol/L)										
Ferritin	0.06 (− 0.59; 0.70)	0.08	0.08	0.05 (− 0.002; 0.90)	0.06	0.08	0.08 (− 0.04; 0.19)	0.06	− 0.02 (− 0.09; 0.04)	0.46
Transferrin	0.004 (− 0.28; 0.27)	0.97	0.32	0.03 (− 0.24; 0.30)	0.97	0.32	0.04 (− 0.38; 0.47)	0.82	0.15 (− 0.18; 0.50)	0.37
TSAT	− 0.04 (− 0.15; 0.05)	0.38	0.26	− 0.02 (− 0.12; 0.08)	0.38	0.26	− 0.04 (− 0.15; 0.09)	0.65	0.03 ( − 020; 0.14)	0.73
Triglyceride (mmol/L)										
Ferritin	0.04 (− 0.01; 0.06)	0.09	0.07	0.02 (− 0.001; 0.07)	0.06	0.11	0.02 (− 0.003; 0.6)	0.18	0.01 (− 0.002; 0.05)	0.28
Transferrin	0.45 (− 0.28; 0.58)	0.07	0.62	0.39 (− 0.16; 0.32)	0.65	0.28	0.35 (− 0.02; 0.47)	0.70	0.55 (− 0.16; 0.60)	0.65
TSAT	− 0.005 (− 0.05; 0.05)	0.85	0.32	− 0.03 (− 0.02; 0.09)	0.22	0.20	− 0.008 (− 0.08; 0.07)	0.22	0.05 (− 0.01; 0.08)	0.33

All iron biomarkers and insulin were log transformed. Model 1 included age. Model 2 included age, BMI, smoking, alcohol use, educational levels, hormone replacement therapy (HRT), antidiabetic and antihypertensive drugs, CVD, and menopause status, when the outcome was insulin or glucose, diabetes was also included in the model. Model 3 stratified by menopause status and included age, BMI, smoking, alcohol use, educational levels, hormone replacement therapy (HRT), antidiabetic and antihypertensive drugs, CVD, and menopause status, when the outcome was insulin or glucose, diabetes was also included in the model. All iron biomarkers were corrected for CRP as suggested by BRINDA protocol prior to analysis. Statistical analysis by multiple linear regression. A negative beta coefficient indicates that the iron biomarker is negatively associated with the levels of the risk factor

HDL-C, High Density lipoprotein cholesterol; LDL: Low-Density Lipoprotein, BMI, body mass index; SBP, systolic blood pressure; DBP, diastolic blood pressure; TC, Total cholesterol; TSAT, transferrin saturation

^§^for interaction between iron marker and menopause

### Variability of outcomes and iron markers

**Fig. 2:**
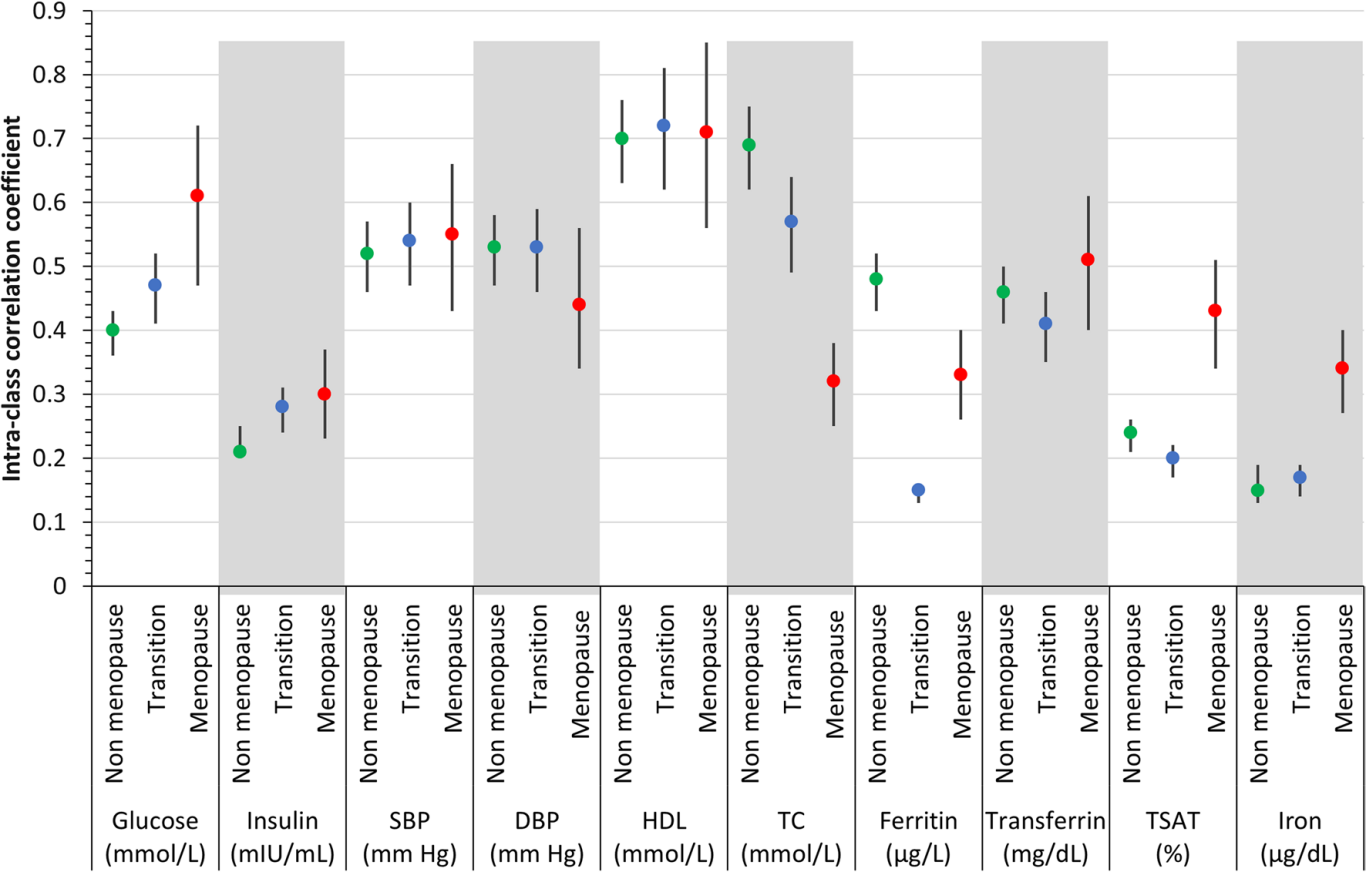
Result of intraclass correlation, stratified by menopausal status, CoLaus study, Laussane, Switzerland. ICC, intra-class correlation coefficient; HDL-C, High density lipoprotein cholesterol; SBP, systolic blood pressure; DBP, diastolic blood pressure; TC: Total cholesterol. Data for 1482 participants. An ICC ≥ 0.75 was considered excellent, between 0.40 and 0.75 good and < 0.40 unsatisfactory

For CVD-RFs, the ICCs varied from 0.32 for insulin to 0.71 for HDL cholesterol (Table 3). Also, except for glucose and total cholesterol, the ICC values did no differ markedly between menopausal categories. All iron biomarkers consistently showed low reliability, especially in the perimenopausal group (Table 3) (Fig. 2),

**Table 3:** Result of intraclass correlation in total longitudinal population (n = 1482), CoLaus study, Lausanne, Switzerland

Variables	ICC (95% CI)
CVD risk factors	
Glucose (mmol/L)	0.60 (0.53, 0.64)
Insulin (mIU/mL)	0.32 (0.29, 0.34)
Systolic blood pressure (mm Hg)	0.59 (0.29, 0.34)
Diastolic blood pressure (mm Hg)	0.49 (0.45, 0.52)
HDL cholesterol (mmol/L)	0.71 (0.65, 0.76)
Total cholesterol (mmol/L)	0.46 (0.42, 0.49)
LDL cholesterol (mmol/L)	0.52 (0.48, 0.55)
Triglyceride (mmol/L)	0.54 (0.43, 0.58)
Iron biomarkers	
Ferritin (μg/L)	0.34 (0.31, 0.36)
Transferrin (mg/dL)	0.47 (0.43, 0.50)
Transferrin saturation (%)	0.32 (0.29, 0.38)
Iron (μg/dL)	0.24 (0.22, 0.28)

Data for 1482 participants. An ICC ≥ 0.75 was considered excellent, between 0.40 and 0.75 good and < 0.40 unsatisfactory

ICC, intra-class correlation coefficient; HDL, high Density lipoprotein cholesterol

### Longitudinal analysis

During the follow-up period, 182 women remained premenopausal, 487 transitioned from premenopause to postmenopause, and 813 remained postmenopausal. The main characteristics of women included according to menopausal status are summarized in Additional file 1: Table S2. The results of Additional file 1: Table S2 reveals differences in various demographic, lifestyle, cardiovascular, and iron biomarker variables among longitudinal participants based on their menopausal status (all p < 0.05). The multivariable linear mixed-model analysis revealed no association (p > 0.05) between changes in iron biomarkers and changes in CVD-RFs during follow-up, except for a negative association between transferrin levels and glucose (− 0.59, 95% CI − 1.10 to − 0.08) and DBP (− 7.81, 95% CI − 15.9 to − 0.56) (Table 4). Additionally, the negative association between transferrin and SBP (p = 0.03) in the age adjusted model, was attenuated (p = 0.11) after adjusting for antihypertensive drugs (Additional file 1: Tables S6 and S6 count).

**Table 4:** Longitudinal association between iron markers and cardiovascular risk factors in the baseline, first and second follow-up of the CoLaus study, Lausanne, Switzerland

Iron Biomarkers	Total, Model 1				Total, Model 2			
	Beta (95% CI)	P-value	P-value ‡	P−value §	Beta (95% CI)	P-value	P-value ‡	P-value §
Sample size	1482				1482			
Glucose (mmol/L)								
Ferritin	0.08 (− 0.001; 0.16)	0.07	0.61	0.93	0.08 (− 0.002; 0.18)	0.07	0.66	0.90
Transferrin	− 0.68 (− 1.16; − 0.20)	0.005	0.91	0.01	− 0.59 (− 1.10; − 0.08)	0.02	0.93	0.07
TSAT	− 0.21 (− 0.38; 0.03)	0.06	0.72	0.01	− 0.11 (− 0.30; 0.06)	0.21	0.82	0.09
Insulin (mIU/mL)								
Ferritin	0.01 (− 0.04; 0.07)	0.53	0.65	0.49	0.03 (− 0.02; 0.09)	0.29	0.25	0.87
Transferrin	− 0.14 (− 0.58; 0.29)	0.52	0.74	0.72	0.07 (− 0.27; 0.42)	0.66	0.70	0.96
TSAT	0.07 ( − 0.04; 0.19)	0.21	0.21	0.70	0.07 (− 0.05; 0.20)	0.26	0.10	0.30
SBP (mm Hg)								
Ferritin	0.21 (− 1.82; 2.25)	0.83	0.68	0.49	0.43 (− 2.05; 2.92)	0.73	0.75	0.72
Transferrin	− 14.8 (− 28.5; − 1.18)	0.03	0.46	0.39	− 10.8 (− 24.1; 2.55)	0.11	0.37	0.43
TSAT	2.41 (− 1.62; 6.45)	0.24	0.99	0.72	2.36 (− 2.55; 7.28)	0.34	0.85	0.71
DBP (mm Hg)								
Ferritin	− 0.02 (− 1.11; 1.05)	0.95	0.94	0.71	0 .23 (− 1.26; 1.73)	0.76	0.77	0.35
Transferrin	− 10.7 (− 19.2; − 2.32)	0.01	0.77	0.20	− 7.81 (− 15.9; − 0.56)	0.04	0.72	0.23
TSAT	0.99 (− 1.14; 3.14)	0.36	0.79	0.26	1.00 (− 1.96; 3.96)	0.50	0.69	0.65
HDL (mmol/L)								
Ferritin	0.03 (− 0.01; 0.07)	0.19	0.65	0.42	0.02 (− 0.03; 0.09)	0.40	0.87	0.31
Transferrin	0.03 (− 0.36; 0.43)	0.86	0.99	0.27	− 0.06 (− 0.43; 0.29)	0.71	0.71	0.15
TSAT	− 0.04 (− 0.13; 0.04)	0.31	0.71	0.77	− 0.02 (− 0.16; 0.10)	0.65	0.88	0.63
TC (mmol/L)								
Ferritin	0.04 (− 0.05; 0.15)	0.34	0.85	0.43	0.07 (− 0.07; 0.21)	0.32	0.71	0.52
Transferrin	0.13 (− 0.65; 0.92)	0.74	0.22	0.004	0.12 (− 0.65; 0.89)	0.75	0.27	0.01
TSAT	0.08 (− 0.11; 0.29)	0.41	0.69	0.83	0.09 (− 0.18; 0.38)	0.51	0.77	0.16
LDL (mmol/L)								
Ferritin	0.06 (− 0.01; 0.07)	0.13	0.69	0.49	0.09 (− 0.02; 1.2)	0.47	0.43	0.50
Transferrin	0.19 (− 0.36; 0.43)	0.89	0.70	0.60	0.14 (− 0.46; 0.33)	0.70	0.71	0.79
TSAT	0.09 (− 0.13; 0.14)	0.33	0.91	0.71	0.08 (− 0.18; 0.21)	0.82	0.89	0.93
Triglyceride (mmol/L)								
Ferritin	0.11 (− 0.03; 0.14)	0.13	0.50	0.33	0.05 (− 0.001; 0.08)	0.19	0.58	0.66
Transferrin	0.21 (− 0.06; 0.30)	0.89	0.86	0.60	0.19 (− 0.03; 0.22)	0.73	0.89	0.52
TSAT	0.06 (− 0.10; 0.14)	0.33	0.35	0.76	0.03 (− 0.01; 0.09)	0.36	0.40	0.32

Iron Biomarkers	Pre-menopause Model 3		Menopause Model 3		Perimenopausal Model 3	
	Beta (95% CI)	P-value	Beta (95% CI)	P-value	Beta (95% CI)	P-value
Sample size	182		813		487	
Glucose (mmol/L)						
Ferritin	0. 05 (− 0.17; 0.29)	0.61	0.16 (− 0.01; 0.32)	0.06	− 0.08 (− 0.24; 0.06)	0.27
Transferrin	− 0.59 (− 1.45; 0.26)	0.17	− 0.98 (− 1.88; − 0.08)	0.03	− 0.12 (− 0.75; 0.50)	0.69
TSAT	− 0.12 (− 0.26; 0.00)	0.06	− 0.14 (− 0.42; 0.13)	0.34	− 0.23 (− 0.53; 0.06)	0.13
Insulin (mIU/mL)						
Ferritin	0.03 (− 0.20; 0.27)	0.77	0.03 (− 0.06; 0.13)	0.50	0.05 (− 0.08; 0.19)	0.41
Transferrin	− 0.37 (− 1.23; 0.49)	0.39	0.48 (− 0.05; 1.03)	0.07	− 0.13 (− 0.66; 0.40)	0.63
TSAT	0.18 (− 0.23; 0.60)	0.38	0.00 (− 0.16; 0.18)	0.91	0.15 (− 0.10; 0.41)	0.23
SBP (mm Hg)						
Ferritin	− 6.61 (− 14.40; 1.17)	0.09	0.34 (− 3.69; 4.37)	0.86	1.00 (− 3.61; 5.62)	0.67
Transferrin	− 13.89 (− 42.36; 14.57)	0.03	− 15.82 (− 38.38; 6.72)	0.16	− 3.18 (− 21.22; 14.86)	0.73
TSAT	− 1.47 (− 15.57; 12.63)	0.83	2.60 (− 4.45; 9.67)	0.46	2.78 (− 5.96; 11.53)	0.53
DBP (mm Hg)						
Ferritin	− 7.71 (− 10.7; 1.27)	0.12	0.72 (− 1.45; 2.91)	0.51	2.11 (− 1.00; 5.23)	0.18
Transferrin	− 6.57 (− 28.75; 15.60)	0.56	− 14.05 (− 26.22; − 1.89)	0.02	− 3.72 (− 15.91; 8.47)	0.55
TSAT	− 2.87 (− 13.74; 8.00)	0.60	2.08 (− 1.73; 5.90)	0.28	1.38 (− 4.53; 7.30)	0.64
HDL (mmol/L)						
Ferritin	0.13 (− 0.11; 0.37)	0.28	0.05 (− 0.04; 0.15)	0.29	− 0.10 (− 0.24; 0.03)	0.13
Transferrin	− 0.17 (− 1.08; 0.73)	0.70	− 0.17 (− 0.73; 0.38)	0.55	− 0.03 (− 0.58; 0.52)	0.91
TSAT	− 0.20 (− 0.64; 0.23)	0.36	0.01 (− 0.16; 0.18)	0.88	− 0.19 (− 0.45; 0.07)	0.16
TC (mmol/L)						
Ferritin	0.01 (− 0.50; 0.54)	0.94	0.13 (− 0.07; 0.35)	0.19	0.02 (− 0.27; 0.32)	0.86
Transferrin	0.58 (− 1.30; 2.48)	0.54	− 0.16 (− 1.33; 1.00)	0.78	− 0.07 (− 1.23; 1.08)	0.90
TSAT LDL (mmol/L)	0.69 (− 0.24; 1.62)	0.14	0.02 (− 0.34; 0.38)	0.09	− 0.11 (− 0.65; 0.47)	0.75
Ferritin	0.08 (− 0.30; 0.19)	0.62	0.09 (− 0.09; 0.22)	0.40	0.04 (− 0.37; 0.14)	0.52
Transferrin	0.28 (− 0.40; 0.68)	0.24	− 0.12 (− 0.83; 0.63)	0.38	− 0.05 (− 1.01; 0.95)	0.46
TSAT	0.42 (− 0.64; 0.92)	0.29	0.32 (− 0.14; 0.60)	0.77	0.21 (− 0.35; 0.67)	0.92
Triglyceride (mmol/L)						
Ferritin	0.07 (− 0.03; 0.25)	0.52	0.05 (− 0.09; 0.19)	0.45	0.03 (− 0.05; 0.11)	0.40
Transferrin	0.20 (− 0.20; 0.30)	0.34	0.15 (− 0.83; 0.23)	0.39	0.13 (− 0.02; 0.16)	0.35
TSAT	0.07 (− 0.05; 0.32)	0.31	0.06 (− 0.14; 0.11)	0.36	0.05 (− 0.01; 0.13)	0.41

All iron biomarkers and insulin were log transformed. Model included age. Model 2 included age, BMI, smoking, alcohol use, educational levels, hormone replacement therapy (HRT), antidiabetic and antihypertensive drugs, CVD, and menopause status, when the outcome was insulin or glucose, diabetes was also included in the model.  Model 3 stratified by menopause status and included age, BMI, smoking, alcohol use, educational levels, hormone replacement therapy (HRT), antidiabetic and antihypertensive drugs, CVD, when the outcome was insulin or glucose, diabetes was also included in the model. All iron biomarkers were corrected for CRP as suggested by BRINDA protocol prior to analysis. Statistical analysis by linear mixed-effect models. The coefficients presented in the table represent the estimated change in the outcome variable (CVD risk factors) associated with a one-unit change in log-transformed iron biomarkers level, after controlling for confounders

HDL-C, High density lipoprotein cholesterol; LDL: BMI, body mass index; SBP, systolic blood pressure; DBP, diastolic blood pressure; TC, Total cholesterol; LDL: Low-Density Lipoprotein, TSAT, transferrin saturation; CVRs, cardiovascular risk factors

^‡^p-value for interaction between iron marker and perimenopause

^§^p-value for interaction between iron marker and menopause

### Sensitivity analysis

Based on our second aim, we examined the interaction between iron biomarker levels and menopausal status in both models 1 and 2. In the cross-sectional analysis, after adjusting for potential confounders, interactions were observed between ferritin and menopausal status (p = 0.03) for HDL cholesterol, and between transferrin and menopausal status for glucose and total cholesterol (p = 0.01, Table 2). In the longitudinal analysis, also adjusted for potential confounders, only an interaction between transferrin and menopausal status for total cholesterol was significant (p = 0.01, Table 4). we stratified cross-sectional and longitudinal analyses by menopausal status to determine if the association between iron biomarkers and CVD risk factors is modified by menopause status. The analyses revealed no changes (p > 0.05) in association (Tables 2 and 4, Additional file 1: Tables S4 to S8). Finally, we categorized iron biomarkers into tertiles and we tested for a linear trend in cross-sectional and longitudinal analyses and the results did not change (Additional file 1: Tables S4 to S8).

## Discussion

This study performed both cross-sectional and prospective analyses to assess the associations between iron biomarkers and CVD-RFs among pre and postmenopausal women. In the cross-sectional analysis, after adjustment for potential confounders, ferritin levels were positively associated with insulin levels, while transferrin levels were positively associated with glucose, insulin, SBP, DBP, triglycerides, total-, LDL- and HDL-cholesterol levels. No association between CVD-RFs and TSAT was found in the fully adjusted model. In the prospective analysis, no association was found between changes of iron biomarkers and changes CVD-RFs during follow up, except for a negative association between changes of transferrin and changes of glucose and DBP.

### Cross-sectional analysis

In line with most previous related studies [21–33], our results indicated that postmenopausal women had higher BMI, CRP, and SBP levels, and were more likely to have CVD and T2D. Moreover, postmenopausal women showed higher levels of ferritin and lower levels of transferrin than premenopausal women [34]. This may be due to menstruation, which causes a loss of ~ 250 mg of iron per year [35]. Serum ferritin levels were positively and independently associated with insulin levels after multivariable adjustment, a finding in agreement with previous studies [36–45]. Because ferritin is also increased in inflammatory status, we included CRP in the multivariable analysis. In agreement with a previous report in women [46], our study shows that ferritin is positively associated with insulin independently of CRP. Iron intervenes in the formation of hydroxyl radicals, which are powerful prooxidants. Therefore, it has been hypothesized that the formation of hydroxyl radicals catalysed by iron contributes to insulin resistance and subsequently to the development of type 2 diabetes [47]. When we stratified by menopausal status, we found significant associations between ferritin and insulin in pre-menopausal women, but not in post-menopausal women, although the confidence intervals overlapped. Previous studies have shown disparity in the association between ferritin and insulin according to sex and menopausal status [37, 40, 48].

Our result indicated that higher levels of transferrin were associated with high levels of glucose, insulin, SBP, DBP and total cholesterol in the fully adjusted model. These findings are in accordance with other studies [49–53]. It is noteworthy to mention that transferrin exhibited a negative correlation with ferritin (r = -0.367, p < 0.001) and total serum iron (TSA) (r = − 0.27, p < 0.001). Conversely, ferritin was positively associated with TSA (r = 0.297, p < 0.001). However, both transferrin and ferritin showed a positive association with insulin.. Our results have confirmed pervious results that showed ferritin and transferrin levels, despite being negatively associated, were independently and positively associated with hyperinsulinemia and hyperglycaemia [49].

No association between CVD-RFs and TSAT was found in the fully adjusted model. It is worth noting that the range of TSAT in our study was (22–36%) which is considered between the normal range among women. A meta-analysis showed a significantly higher risk of T2D for a TSAT ≥ 50%, which is above the normal range (20–50%) and thus suggestive of iron overload [54].

### Low reliability of iron biomarkers and pitfalls of cross-sectional design

In our study all iron biomarkers (serum Iron, serum ferritin, transferrin and TSAT) had a low reliability as defined by the ICC, especially in the perimenopausal group. Our findings are in line with a Dutch study that assessed the reliability of 20 serum biomarkers collected several years apart in men: except for ferritin, the other iron-related biomarkers had poor reliability [55]. By using unreliable biomarkers as a one-time exposure in cohort studies, the exposure- disease associations are likely to be lower than the true associations due to external variables and biological variability [56]. To mitigate these challenges, in our study we took advantage of repeated measures of iron biomarkers to correct for this low reliability and improve precision and the ability to capture changes over time.

### Longitudinal analysis

Our results showed only a negative association between changes of transferrin and glucose and diastolic blood pressure. Our results do not replicate those of a Chinese study including 8337 adults, where transferrin levels were positively associated with incident hypertension [57]. Our results also do not replicate those of a prospective study where serum transferrin was inversely associated with insulin resistance and baseline serum ferritin, transferrin, and total serum iron were significantly associated with incident T2DM [58]. The low reliability of iron markers as described above might partly explain the lack of association between iron markers and CVD-RFs in our study. Our results indicated that due to menopause-induced changes in iron levels, one single measure of iron biomarkers might not be enough when exploring longitudinal associations, including the association of iron biomarkers with cardiometabolic risk factors changes, and that a prospective setting might be more robust than the cross-sectional one. Furthermore, our findings showed that different iron biomarkers exhibit inconsistent associations with CVD-RFs, independently of potential confounding factors. This implies that the exploration of the association between iron and CVD-RFs may require the utilization of different iron biomarkers (such as ferritin, transferrin, transferrin saturation, and so on). Also, as most of the cross-sectional associations we found did not hold in the longitudinal analysis, future studies exploring the association between iron and cardiometabolic risk should be prospective rather than cross-sectional.

Carrying out reliability studies before performing expensive analyses of biomarkers are necessary to investigate which biomarkers are less likely to cause attenuation of observed exposure outcome associations due to inter and between-individual variability. Our results support the importance of using repeated measurements of iron biomarkers instead of relying on single measurements, particularly in the context of low reliability. By incorporating multiple measurements, researchers can improve the stability, accuracy, reproducibility, and precision of iron biomarker assessments, leading to more reliable diagnoses, monitoring, and evaluation of interventions related to iron status.

### Strengths and limitations

This study highlighted the significance of repeated measurements for minimizing the impact of measurement variability and increasing the reliability of iron biomarker evaluations. We used repeated measurements of iron biomarkers to assess the associations between iron biomarkers and changes in CVD-RFs, while most of previous prospective studies used a single measurement. Repeated individual assessments of iron biomarkers decrease the impact of intra-individual variability. Also using of both cross-sectional and prospective design, a relatively large sample size and the use of several markers of iron metabolism, contrary to other studies that focused only on ferritin or ferritin and transferrin [59].

Some limitation needs to be addressed. First, a sizable portion of the initial cohort was excluded from the analysis. This procedure may have favored the selection of the most motivated individuals (with complete data and follow-ups), potentially introducing selection bias.. Second, the study was conducted at the single location in Switzerland, using a very ethnically limited cohort so results might not be generalizable to other settings. Third, the natural variability of some CVD risk factors such as blood pressure and to a lesser degree of lipid and glucose markers might reduce the association. A better strategy would be to consider CVD events, but even those are subject to possible misdiagnosis due to lack of adequate information for correct adjudication as recommended [60]. Lastly, it would have been interesting to report the magnitude of the relationships between iron markers and cardiometabolic risk factors. Still, as all associations were assessed using log-transformed data, interpreting the relationships would be complicated as back-transformation would be needed, and the linearity of the relationships would no longer hold.

## Conclusion

In cross-sectional analysis, transferrin was associated with several CVD-RFs, and the associations did not change according to menopausal status. Conversely, in the longitudinal analyses, transferrin was associated only with lower glucose and diastolic blood pressure levels. These differences might stem from the substantial longitudinal variation of iron biomarkers. Hence, prospective studies with multiple measurements of iron markers should be preferred to cross-sectional analyses.

### Supplementary Information

**Additional file 1: Table S1.** Comparison between Included and Excluded participants CoLaus study, Lausanne, Switzerland. **Table S2.** Characteristic of the study of longitudinal participants, CoLaus study, Lausanne, Switzerland. **Table S3.** P-values for the quadratic and cubic terms of iron biomarkers, regression analysis of the associations between iron biomarkers and cardiovascular risk factors at baseline, CoLaus study, Lausanne, Switzerland. **Table S4.** Results of the associations between log-transformed ferritin levels and CVD risk factors at baseline (2003-2006), CoLaus study, Lausanne, Switzerland. **Table S5.** Results of the associations between log-transformed transferrin levels and CVD risk factors at baseline (2003-2006), CoLaus study, Lausanne, Switzerland. **Table S6.** Checking which variable cancels the longitudinal association between log-transformed transferrin levels and systolic blood pressure in total population (n= 1482), CoLaus study, Lausanne, Switzerland. **Table S7. **Longitudinal associations between log-transformed ferritin levels and CVD risk factors in the baseline, first, and second follow-ups of the CoLaus study, Lausanne, Switzerland. **Table S8.** Longitudinal associations between log-transformed transferrin levels and CVD risk factors in the baseline, first, and second follow-ups of the CoLaus study, Lausanne, Switzerland. **Table S9. **Longitudinal associations between log-transformed transferrin saturation levels and CVD risk factors in the baseline, first, and second follow-ups of the CoLaus study, Lausanne, Switzerland.

## Data Availability

The data of CoLaus|PsyCoLaus study used in this article cannot be fully shared as they contain potentially sensitive personal information on participants. According to the Ethics Committee for Research of the Canton of Vaud, sharing these data would be a violation of Swiss legislation with respect to privacy protection. However, coded individual-level data that do not allow researchers to identify participants are available upon request to researchers who meet the criteria for data sharing of the CoLaus|PsyCoLaus Datacenter (CHUV, Lausanne, Switzerland). Any researcher affiliated to a public or private research institution who complies with the CoLaus|PsyCoLaus standards can submit a research application to research.colaus@chuv.ch or research.psycolaus@chuv.ch. Proposals requiring baseline data only, will be evaluated by the baseline (local) Scientific Committee (SC) of the CoLaus and PsyCoLaus studies. Proposals requiring follow-up data will be evaluated by the follow-up (multicentric) SC of the CoLaus|PsyCoLaus cohort study. Detailed instructions for gaining access to the CoLaus|PsyCoLaus data used in this study are available at www.colaus-psycolaus.ch/professionals/how-to-collaborate/.
