# Supplementary material for: Cross-sectional and longitudinal associations of Iron biomarkers and cardiovascular risk factors in pre- and postmenopausal women: leveraging repeated measurements to address natural variability
Source: Cardiovasc Diabetol. 2024 May 7;23:158. doi: 10.1186/s12933-024-02242-x (PMC11077797; doi:10.1186/s12933-024-02242-x)
Supplement: Supplementary file 1 — Additional file 1: Table S1. Comparison between Included and Excluded participants CoLaus study, Lausanne, Switzerland. Table S2. Characteristic of the study of longitudinal participants, CoLaus study, Lausanne, Switzerland. Table S3. P-values for the quadratic and cubic terms of iron biomarkers, regression analysis of the associations between iron biomarkers and cardiovascular risk factors at baseline, CoLaus study, Lausanne, Switzerland. Table S4. Results of the associations between log-transformed ferritin levels and CVD risk factors at baseline (2003-2006), CoLaus study, Lausanne, Switzerland. Table S5. Results of the associations between log-transformed transferrin levels and CVD risk factors at baseline (2003-2006), CoLaus study, Lausanne, Switzerland. Table S6. Checking which variable cancels the longitudinal association between log-transformed transferrin levels and systolic blood pressure in total population (n= 1482), CoLaus study, Lausanne, Switzerland. Table S7. Longitudinal associations between log-transformed ferritin levels and CVD risk factors in the baseline, first, and second follow-ups of the CoLaus study, Lausanne, Switzerland. Table S8. Longitudinal associations between log-transformed transferrin levels and CVD risk factors in the baseline, first, and second follow-ups of the CoLaus study, Lausanne, Switzerland. Table S9. Longitudinal associations between log-transformed transferrin saturation levels and CVD risk factors in the baseline, first, and second follow-ups of the CoLaus study, Lausanne, Switzerland. [file 12933_2024_2242_MOESM1_ESM.docx]

**Additional information**

**Table S1.** Comparison between Included and Excluded participants CoLaus study, Lausanne, Switzerland.

| **Variable** | **Included** | **Excluded** | **P value*** |
| --- | --- | --- | --- |
| Sample size | 2542 | 1002 |  |
| Age (years) | 53.2 (10.4) | 51.3 (10.5) | <0.001 |
| Smoking status (n, %) |  |  | 0.43 |
| Never | 1192 (46.8) | 472 (47.1) |  |
| Former | 793 (31.2) | 254 (25.3) |  |
| Current | 557 (21.9) | 276 (27.5) |  |
| Education level (n, %) |  |  | 0.31 |
| High | 420 (16.5) | 196 (19.5) |  |
| Middle | 688 (27.1) | 263 (26.3) |  |
| Low | 1434 (56.4) | 543 (54.2) |  |
| Use of antihypertensive drugs (n, %) | 404 (15.8) | 150 (14.7) | 0.42 |
| Use of antidiabetic drugs (n, %) | 53 (2.1) | 39 (3.8) | 0.02 |
| Alcohol drinker | 1,611 (63.3) | 574 (57.2) | 0.01 |
| Body mass index (kg/m2) | 25.2 (4.6) | 24.8 (5.2) | 0.03 |
| High-sensitivity C-reactive protein, mg/l | 1.3 (0.6-2.7) | 1.3 (0.6-4.4) | 0.06 |
| Prevalence of CVD | 125 (4.9) | 48 (4.3) | 0.83 |
| Prevalence of diabetes | 94 (3.7) | 48 (4.7) | 0.14 |
| Cardiovascular risk factors |  |  |  |
| Glucose (mmol/L) | 5.30 (0.7) | 5.28 (1.1) | 0.06 |
| Insulin (mIU/mL) | 6.01 (4.4-9.5) | 6.11 (4.4-9.4) | 0.08 |
| SBP (mm Hg) | 124 (17) | 123.9 (18.1) | 0.06 |
| DBP (mm Hg) | 77 (11) | 77.4 (10.9) | 0.08 |
| HDL-C (mmol/L) | 1.80 (0.4) | 1.82 (0.41) | 0.07 |
| TC (mmol/L) | 5.62 (1.02) | 5.48 (0.99) | 0.28 |
| LDL (mmol/L) | 3.30 (0.9) | 3.19 (0.8) | 0.06 |
| Triglyceride (mmol/L) | 1 (0.8-1.4)) | 0.9 (0.8-1.3) | 0.07 |
| Iron biomarkers |  |  |  |
| Iron (μg/dL) | 95 (74-119) | 93 (71-114) | 0.05 |
| Ferritin ( μg/L) | 78 (44-138) | 77 (43-126) | 0.06 |
| Transferrin (mg/dL) | 236 (213-263) | 234 (206-259) | 0.07 |
| Transferrin saturation (%) | 29.5 (22.4-36.6) | 27.9 (20.4-33.4) | 0.06 |

Continuous variables shown as mean (SD) with p according to t-test; categorical variables as % with p according to χ2, median (25th-75th percentile) with p according to Mann–Whitney U-test. CVDRFs: Cardiovascular disease risk factors, CVD: Cardiovascular disease, HDL-C: High density lipoprotein cholesterol, BMI, body mass index. SBP: systolic blood pressure, DBP: diastolic blood pressure, TC: Total cholesterol, * Compare the characteristics of women respondents who were included versus those who were excluded.

**Table S2. Characteristic of the study** of **longitudinal participants, CoLaus study, Lausanne, Switzerland.**

| **Variable** | **Total** | **Pre-menopause** | **Perimenopausal** | **Menopause** | **P value*** |
| --- | --- | --- | --- | --- | --- |
| Sample size | 1482 | 182 | 487 | 813 |  |
| Age (years) | 53.4 (10.3) | 38.9 (2.6) | 46.1(4.6) | 60.9 (6.5) | <0.001 |
| Smoking status (n, %) |  |  |  |  | <0.001 |
| Never | 682 (46.02) | 95 (52.2) | 177 (36.3) | 410 (50.4) |  |
| Former | 473 (31.9) | 44 (24.1) | 172 (35.3) | 257 (31.6) |  |
| Current | 327 (22.06) | 43 (23.6) | 138 (28.3) | 146 (18.0) |  |
| Education level (n, %) |  |  |  |  | <0.001 |
| High | 248 (16.7) | 50 (27.4) | 103 (21.2) | 95 (11.4) |  |
| Middle | 408 (27.5) | 62 (34.1) | 137 (28.1) | 209 (25.8) |  |
| Low | 826 (55.7) | 70 (38.4) | 247 (50.7) | 509 (62.7) |  |
| Antihypertensive drugs (n, %) | 199 (13.4) | 0 (0.00) | 37 (7.5) | 162 (19.9) | <0.001 |
| Alcohol drinker (%) | 1,011 (68.3) | 121 (66.9) | 344 (70.9) | 546 (67.1) | 0.03 |
| Body mass index (kg/m2) | 24.7 (4.2) | 23.6 (3.9) | 23.9 (3.9) | 25.6 (4.9) | 0.02 |
| High-sensitivity C-reactive protein, (mg/l) | 1.2 (0.6-2.4) | 0.8 (0.4- 2.1) | 0.9 (0.4- 1.9) | 1.5 (0.7- 2.6) | 0.04 |
| Prevalence of CVD (n, %) | 82 (5.5) | 3 (1.6) | 25 (5.13) | 54 (6.6) | 0.02 |
| Prevalence of diabetes | 40 (2.7) | 0 (0.00) | 10 (2.05) | 30 (3.6) | 0.04 |
| Antidiabetic drugs (n, %) | 23 (1.5) | 0 (00.0) | 1 (0.21) | 22 (2.7) | <0.001 |
| Cardiovascular risk factors |  |  |  |  |  |
| SBP (mm Hg) | 124 (17.6) | 114 (12) | 117 (13) | 130 (19) | 0.01 |
| DBP (mm Hg) | 77 (10) | 74 (10) | 76 (11) | 78 (11) | 0.02 |
| Glucose (mmol/L) | 5.31 (0.7) | 5.1 (0.4) | 5.2 (0.4) | 5.4 (0.9) | 0.03 |
| Insulin (mIU/mL) | 6.01 (4.4-9.5) | 6.57 (4.6-9.8) | 5.63 (4 - 8) | 6.45 (4.6-9.6) | 0.03 |
| HDL-C (mmol/L) | 1.81 (0.42) | 1.73 (0.33) | 1.82 (0.42) | 1.80 (0.44) | 0.04 |
| TC (mmol/L) | 5.65 (1.1) | 4.95 (0.80) | 5.21 (0.91) | 5.99 (0.96) | 0.01 |
| LDL (mmol/L)  Triglyceride (mmol/L) | 3.38 (0.94)  1.1 (0.8-1.3) | 3.1 (0.81)  0.8 (0.7-1.4) | 3.3 (96)  0.9 (0.7-1.3) | 3.4 (0.91)  1.1 (0.8-1.4) | 0.04  0.04 |
| Iron biomarkers |  |  |  |  |  |
| Iron (μg/dL) | 96 (76-119) | 91 (72-116) | 97(72-123) | 97 (76-118) | 0.03 |
| Ferritin (μg/L) | 79 (43-140) | 53 (32-82) | 54 (30-89) | 112 (66-180) | 0.004 |
| Transferrin (mg/dL) | 237 (213-263) | 239 (216-276) | 239 (215-270) | 232(211-276) | 0.04 |
| Transferrin saturation (%) | 29.6 (22.4-36.6) | 27.1 (20.4-35.4) | 29.1 (21.4-35.4) | 30.1 (23.4-36.4) | 0.03 |

CVD-RFs : Cardiovascular disease risk factors, CVD: Cardiovascular disease, HDL-C: High density lipoprotein cholesterol, LDL : Low-Density Lipoprotein , BMI, body mass index. SBP, systolic blood pressure; DBP, diastolic blood pressure; TC: Total cholesterol. Continuous variables shown as mean (SD) or median (25^th^-75^th^ percentile); categorical variables show as %. Comparisons performed between menopausal categories using ANOVA or Kruskal-Wallis test for continuous variables and χ2 or Fisher’s exact test for categorical variables.

**able S3.** P-values for the quadratic and cubic terms of iron biomarkers, regression analysis of the associations between iron biomarkers and cardiovascular risk factors at baseline, CoLaus study, Lausanne, Switzerland.

|  | **Model 1 Model 2** | | **Model 1** | **Model 2** |
| --- | --- | --- | --- | --- |
|  | **Quadratic** | **Quadratic** | **Cubic** | **Cubic** |
| Sample size |  |  |  |  |
| **Glucose (mmol/L)** |  |  |  |  |
| Ferritin | 0.18 | 0.11 | 0.06 | 0.16 |
| Transferrin | 0.06 | 0.05 | 0.09 | 0.08 |
| TSAT | 0.08 | 0.35 | 0.08 | 0.49 |
| **Insulin (mIU/mL)** |  |  |  |  |
| Ferritin | 0.23 | 0.17 | 0.08 | 0.28 |
| Transferrin | 0.81 | 0.72 | 0.58 | 0.39 |
| TSAT | 0.07 | 0.21 | 0.09 | 0.22 |
| **SBP (mm Hg)** |  |  |  |  |
| Ferritin | 0.22 | 0.22 | 0.09 | 0.18 |
| Transferrin | 0.92 | 0.90 | 0.77 | 0.72 |
| TSAT | 0.08 | 0.06 | 0.08 | 0.07 |
| **DBP (mm Hg)** |  |  |  |  |
| Ferritin | 0.37 | 0.38 | 0. 11 | 0.39 |
| Transferrin | 0.82 | 0.74 | 0.63 | 0.47 |
| TSAT | 0.09 | 0.72 | 0.06 | 0.53 |
| **HDL (mmol/L)** |  |  |  |  |
| Ferritin | 0.52 | 0.42 | 0.56 | 0.59 |
| Transferrin | 0.39 | 0.30 | 0.21 | 0.31 |
| TSAT | 0.52 | 0.11 | 0.16 | 0.33 |
| **TC (mmol/L)** |  |  |  |  |
| Ferritin | 0.68 | 0.71 | 0.42 | 0.63 |
| Transferrin | 0.39 | 0.36 | 0.42 | 0.33 |
| TSAT  **LDL (mmol/L)**  Ferritin  Transferrin  TSAT  **Triglyceride (mmol/L)**  Ferritin  Transferrin  TSAT | 0.48  0.50  0.40  0.06  0.52  0.48  0.09 | 0.09  0.71  0.36  0.09  0.58  0.30  0.19 | 0.22  0.73  0.46  0.10  0.98  0.36  0.22 | 0.20  0.80  0.32  0.13  0.69  0.35  0.39 |

HDL-C, High Density lipoprotein cholesterol; BMI, body mass index; SBP, systolic blood pressure; DBP, diastolic blood pressure; TC, Total cholesterol; TSAT, transferrin saturation. All iron biomarkers and insulin were log transformed. Model 1 included age. Model 2 included age, BMI, smoking, alcohol use, educational levels, hormone replacement therapy (HRT), antidiabetic and antihypertensive drugs, CVD, and menopause status, when the outcome was insulin or glucose, diabetes was also included in the model. All iron biomarkers were corrected for CRP as suggested by BRINDA protocol prior to analysis. Statistical analysis by multiple linear regression.

**Table S4**. Results of the associations between log-transformed ferritin levels and CVD risk factors at baseline (2003-2006), CoLaus study, Lausanne, Switzerland.

| **Variable** | **Total, Model 1**  **Beta (95% CI)** | **P-value** | **Total, Model 2**  **Beta (95% CI)** | **P-value** | **Menopause**  **Model 3**  **Beta (95% CI)** | **P-value** | **Pre-menopause Model 3**  **Beta (95% CI)** | **P value** |
| --- | --- | --- | --- | --- | --- | --- | --- | --- |
| **Sample size** | 2542 |  | 2542 |  | 1421 |  | 1121 |  |
| **Glucose (mmol/L)** |  |  |  |  |  |  |  |  |
| T1 | Reference |  | Reference |  | Reference |  | Reference |  |
| T2 | -0.01 (-0.10; 0.08) | 0.80 | 0.00 (-0.06; 0.08) | 0.80 | -0.06 (-0.20; 0.07) | 0.37 | 0.04 (-0.02; 0.12) | 0.22 |
| T3 | 0.11 (0.01; 0.21) | 0.02 | 0.07 (-0.00; 0.15) | 0.07 | -0.00 (-0.13; 0.12) | 0.93 | 0.11 (0.01; 0.21) | 0.01 |
| p for trend | 0.03 |  | 0.07 |  | 0.81 |  | 0.01 |  |
| **Insulin (mIU/mL)** |  |  |  |  |  |  |  |  |
| T1 | Reference |  | Reference |  | Reference |  | Reference |  |
| T2 | 0.07 (0.00; 0.14) | 0.04 | 0.02 (-0.03; 0.08) | 0.33 | 0.01 (-0.07; 0.11) | 0.68 | 0.02 (-0.05; 0.10) | 0.54 |
| T3 | 0.12 (0.05; 0.20) | 0.00 | 0.07 (0.01; 0.14) | 0.01 | 0.05 (-0.03; 0.14) | 0.22 | 0.09 (-0.01; 0.19) | 0.08 |
| p for trend | 0.00 |  | 0.01 |  | 0.19 |  | 0.09 |  |
| **SBP (mm Hg)** |  |  |  |  |  |  |  |  |
| T1 | Reference |  | Reference |  | Reference |  | Reference |  |
| T2 | -0.70 (-2.68; 1.27) | 0.48 | -0.81 (-2.77; 1.15) | 0.41 | -3.65 (-7.08; -0.23) | 0.03 | 1.18 (-1.02; 3.38) | 0.29 |
| T3 | -0.16 (-2.25; 1.92) | 0.87 | -0.36 (-2.44; 1.72) | 0.73 | -2.49 (-5.74; 0.75) | 0.13 | 1.60 (-1.27; 4.49) | 0.27 |
| p for trend | 0.87 |  | 0.72 |  | 0.29 |  | 0.19 |  |
| **DBP (mm Hg)** |  |  |  |  |  |  |  |  |
| T1 | Reference |  | Reference |  | Reference |  | Reference |  |
| T2 | -0.14 (-1.46; 1.17) | 0.83 | -0.32 (-1.61; 0.96) | 0.61 | -1.26 (-3.36; 0.82) | 0.23 | -0.14 (-1.77; 1.48) | 0.86 |
| T3 | 0.01 (-1.37; 1.41) | 0.97 | -0.33 (-1.69; 1.03) | 0.63 | -1.18 (-3.17; 0.79) | 0.24 | 0.06 (-2.06; 2.20) | 0.95 |
| p for trend | 0.98 |  | 0.63 |  | 0.32 |  | 0.99 |  |
| **HDL (mmol/L)** |  |  |  |  |  |  |  |  |
| T1 | Reference |  | Reference |  | Reference |  | Reference |  |
| T2 | -0.01 (-0.07; 0.04) | 0.59 | -0.01 (-0.06; 0.03) | 0.54 | -0.03 (-0.11; 0.05) | 0.45 | -0.02 (-0.09; 0.04) | 0.48 |
| T3 | -0.01 (-0.07; 0.04) | 0.63 | -0.00 (-0.06; 0.04) | 0.77 | -0.04 (-0.12; 0.03) | 0.22 | 0.05 (-0.02; 0.14) | 0.19 |
| p for trend | 0.63 |  | 0.77 |  | 0.23 |  | 0.40 |  |
| **TC (mmol/L)** |  |  |  |  |  |  |  |  |
| T1 | Reference |  | Reference |  | Reference |  | Reference |  |
| T2 | 0.09 (-0.03; 0.21) | 0.15 | 0.06 (-0.05; 0.19) | 0.28 | 0.11 (-0.09; 0.32) | 0.26 | -0.00 (-0.15; 0.14) | 0.94 |
| T3 | 0.06 (-0.06; 0.19) | 0.34 | 0.04 (-0.08; 0.17) | 0.49 | 0.09 (-0.10; 0.28) | 0.36 | -0.05 (-0.24; 0.14) | 0.58 |
| p for trend | 0.34 |  | 0.48 |  | 0.48 |  | 0.63 |  |

HDL-C, High-density lipoprotein cholesterol; BMI, body mass index; SBP, systolic blood pressure; DBP, diastolic blood pressure; TC, Total cholesterol; TSAT, Transferrin saturation. All iron biomarkers and insulin were log-transformed. Model 1 included age. Model 2 included age, BMI, smoking, alcohol use, educational levels, hormone replacement therapy (HRT), antidiabetic and antihypertensive drugs, CVD, and menopause status, when the outcome was insulin or glucose, diabetes was also included in the model. Model 3 stratified by menopause status and included age, BMI, smoking, alcohol use, educational levels, hormone replacement therapy (HRT), antidiabetic and antihypertensive drugs, CVD, when the outcome was insulin or glucose, diabetes was also included in the model. All iron biomarkers were corrected for CRP as suggested by BRINDA protocol prior to analysis. Statistical analysis by multiple linear regression.

**Table S5**. Results of the associations between log-transformed transferrin levels and CVD risk factors at baseline (2003-2006), CoLaus study, Lausanne, Switzerland.

| **Variable** | **Total**  **Model 1**  **Beta (95% CI)** | **P-value** | **Total**  **Model 2**  **Beta (95% CI)** | **P-value** | **Menopause**  **Model 3**  **Beta (95% CI)** | **P-value** | **Pre-menopause Model 3**  **Beta (95% CI)** | **P-value** |
| --- | --- | --- | --- | --- | --- | --- | --- | --- |
| **Sample size** | 2542 |  | 2542 |  | 1421 |  | 1121 |  |
| **Glucose (mmol/L)** |  |  |  |  |  |  |  |  |
| T1 | Reference |  | Reference |  | Reference |  | Reference |  |
| T2 | 0.02 (-0.07; 0.11) | 0.65 | -0.00 (-0.08; 0.06) | 0.81 | -0.03 (-0.14; 0.07) | 0.53 | 0.03 (-0.04; 0.12) | 0.38 |
| T3 | 0.11 (0.01; 0.20) | 0.02 | 0.03 (-0.04; 0.10) | 0.40 | 0.08 (-0.04; 0.20) | 0.19 | 0.00 (-0.07; 0.08) | 0.88 |
| p for trend | 0.02 |  | 0.40 |  | 0.22 |  | 0.93 |  |
| **Insulin (mIU/mL)** |  |  |  |  |  |  |  |  |
| T1 | Reference |  | Reference |  | Reference |  | Reference |  |
| T2 | 0.04 (-0.03; 0.11) | 0.25 | 0.02 (-0.02; 0.08) | 0.32 | 0.03 (-0.03; 0.11) | 0.34 | 0.03 (-0.06; 0.12) | 0.52 |
| T3 | 0.11 (0.04; 0.18) | 0.00 | 0.15 (0.09; 0.21) | 0.00 | 0.17 (0.09; 0.25) | 0.00 | 0.15 (0.06; 0.23) | 0.00 |
| p for trend | <0.001 |  | <0.001 |  | <0.001 |  | <0.001 |  |
| **SBP (mm Hg)** |  |  |  |  |  |  |  |  |
| T1 | Reference |  | Reference |  | Reference |  | Reference |  |
| T2 | 1.27 (-0.66; 3.21) | 0.19 | 1.32 (-0.59; 3.24) | 0.17 | 0.11 (-2.66; 2.90) | 0.93 | 3.24 (0.71; 5.76) | 0.01 |
| T3 | 4.24 (2.29; 6.18) | 0.00 | 4.14 (2.21; 6.07) | 0.00 | 3.74 (0.76; 6.72) | 0.01 | 4.64 (2.26; 7.01) | 0.00 |
| p for trend | <0.001 |  | <0.001 |  | 0.01 |  | <0.001 |  |
| **DBP (mm Hg)** |  |  |  |  |  |  |  |  |
| T1 | Reference |  | Reference |  | Reference |  | Reference |  |
| T2 | 0.78 (-0.50; 2.08) | 0.23 | 0.91 (-0.34; 2.17) | 0.15 | 0.83 (-0.86; 2.53) | 0.33 | 0.93 (-0.93; 2.80) | 0.32 |
| T3 | 2.37 (1.07; 3.67) | 0.00 | 2.42 (1.82; 3.69) | 0.00 | 1.97 (0.15; 3.80) | 0.03 | 3.01 (1.25; 4.77) | 0.00 |
| p for trend | <0.001 |  | <0.001 |  | 0.03 |  | <0.001 |  |
| **HDL (mmol/L)** |  |  |  |  |  |  |  |  |
| T1 | Reference |  | Reference |  | Reference |  | Reference |  |
| T2 | -0.00 (-0.06; 0.04) | 0.78 | -0.00 (-0.06; 0.04) | 0.72 | -0.02 (-0.09; 0.03) | 0.40 | 0.01 (-0.06; 0.09) | 0.68 |
| T3 | 0.00 (-0.05; 0.05) | 0.92 | 0.01 (-0.04; 0.06) | 0.69 | -0.02 (-0.09; 0.04) | 0.51 | 0.05 (0.02; 0.12) | 0.16 |
| p for trend | 0.92 |  | 0.69 |  | 0.48 |  | 0.15 |  |
| **TC (mmol/L)** |  |  |  |  |  |  |  |  |
| T1 | Reference |  | Reference |  | Reference |  | Reference |  |
| T2 | -0.02 (-0.15; 0.09) | 0.66 | -0.01 (-0.14; 0.10) | 0.76 | -0.06 (-0.23; 0.10) | 0.45 | 0.02 (-0.14; 0.19) | 0.77 |
| T3 | -0.02 (-0.14; 0.10) | 0.72 | 0.00 (-0.12; 0.12) | 0.99 | -0.13 (-0.31; 0.04) | 0.15 | 0.17 (0.01; 0.33) | 0.03 |
| p for trend | 0.72 |  | 0.99 |  | 0.15 |  | 0.03 |  |

HDL-C, High-density lipoprotein cholesterol; BMI, body mass index; SBP, systolic blood pressure; DBP, diastolic blood pressure; TC, Total cholesterol; TSAT, Transferrin saturation. Model 1 included age. Model 2 included age, BMI, smoking, alcohol use, educational levels, hormone replacement therapy (HRT), antidiabetic and antihypertensive drugs, CVD, and menopause status, when the outcome was insulin or glucose, diabetes was also included in the model. Model 3 stratified by menopause status and included age, BMI, smoking, alcohol use, hormone replacement therapy (HRT), antidiabetic and antihypertensive drugs, CVD, and menopause status, when the outcome was insulin or glucose, diabetes was also included in the model. All iron biomarkers were corrected for CRP as suggested by BRINDA protocol prior to analysis. Statistical analysis by multiple linear regression.

**Table S6**. Checking which variable cancels the longitudinal association between log-transformed transferrin levels and systolic blood pressure in total population (n= 1482), CoLaus study, Lausanne, Switzerland.

|  | **Beta (95% CI)** | **P-value** | **Beta (95% CI)** | **P-value** | **Beta (95% CI)** | **P-value** | **Beta (95% CI)** | **P-value** |
| --- | --- | --- | --- | --- | --- | --- | --- | --- |
| Transferrin | -14.8 (-28.5; -1.18) | 0.03 | -12.3 (-23.1; -1.55) | 0.02 | -10.1 (-0.21; -0.11) | 0.04 | -10.8 (-24.1; -0.04) | 0.04 |
| Age | 0.73 (0.70; 0.82) | <0.001 | 0.71 (0.66; 0.77) | <0.001 | 0.69 (0.64; 074) | <0.001 | 0.69 (0.64; 0.74) | <0.001 |
| BMI |  |  | 0.57 (0.44; 0.70) | <0.001 | 0.53 (0.40; 0.66) | <0.001 | 0.52 (0.35; 0.65) | 0.04 |
| Smoking |  |  |  |  | 0.62 (0.60; 0.70) | 0.007 | 0.60 (0.50; 0.69) | 0.03 |
| Alcohol use |  |  |  |  |  |  | 0.49 (-0.03; 0.65) | 0.40 |
| HRT |  |  |  |  |  |  |  |  |
| Education level |  |  |  |  |  |  |  |  |
| CVD |  |  |  |  |  |  |  |  |
| Anti-hypertensive |  |  |  |  |  |  |  |  |
| Menopause status |  |  |  |  |  |  |  |  |

BMI, body mass index; CVD, cardiovascular disease; HRT, hormone replacement therapy. Transferrin levels were corrected for CRP as suggested by BRINDA protocol prior to analysis. Statistical analysis by linear mixed-effect models.

**Table S6(cont.)**. Checking which variable cancels the longitudinal association between log-transformed transferrin levels and systolic blood pressure in total population (n = 1482), CoLaus study, Lausanne, Switzerland.

|  | **Beta (95% CI)** | **P-value** | **Beta (95% CI)** | **P-value** | **Beta (95% CI)** | **P-value** | **Beta (95% CI)** | **P-value** | **Beta (95% CI)** | **P-value** |
| --- | --- | --- | --- | --- | --- | --- | --- | --- | --- | --- |
| Transferrin | -12.3 (-23.5; -1.46) | 0.02 | -12.6 (-23.1; -1.71) | 0.02 | -11.1 (-0.21; -0.31) | 0.04 | -10.9 (-25.1; 2.57) | 0.07 | -10.8 (-24.1; 2.55) | 0.11 |
| Age | 0.71 (0.65; 0.69) | <0.001 | 0.69 (0.52; 0.75) | <0.001 | 0.67 (0.61; 073) | <0.001 | 0.52 (0.43; 0.61) | 0.002 | 0.50 (0.43; 0.59) | <0.001 |
| BMI | 0.57 (0.44; 0.70) | <0.001 | 0.52 (0.44; 0.65) | <0.001 | 0.48 (0.35; 0.61) | <0.001 | 0.34 (0.25; 0.52) | 0.03 | 0.33 (0.20; 0.47) | 0.04 |
| Smoking | 0.59 (0.48; 0.68) | 0.02 | 0.56 (0.49; 0.63) | 0.02 | 0.50 (0.43; 0.56) | 0.03 | 0.48 (0.44; 0.55) | 0.04 | 0.46 (0.42; 0.50) | 0.04 |
| Alcohol use | 0.46 (-0.02; 0.53) | 0.08 | 0.42 (-0.02; 0.49) | 0.32 | 0.40 (-0.01; 0.43) | 0.39 | 0.36 (-0.03; 0.44) | 0.55 | 0.30 (-0.01; 0.40) | 0.62 |
| HRT | -0.05(-1.02;1.23) | 0.93 | -0.07 (-1.32; 1.24) | 0.90 | -0.08 (-1.39; 1.23) | 0.92 | -0.27 (-1.61; 1.13) | 0.68 | -0.20 (-1.69; 1.19) | 0.78 |
| Education level |  |  | 1.32 (0.63; 2.15) | 0.001 | 1.2 (0.64; 2.14) | 0.002 | 1.1 (0.54; 2.10) | 0.001 | 1.06 (0.44; 2.05) | 0.005 |
| CVD |  |  |  |  | 1.8 (-1.45; 4.21) | 0.22 | 0.52 (-2.33; 3.41) | 0.69 | 0.58(-2.30; 3.20) | 0. 85 |
| Anti-hypertensive |  |  |  |  |  |  | 5.60 (5.05; 8.41) | 0.001 | 5.30 (4.95; 7.41) | 0.003 |
| Menopause status |  |  |  |  |  |  |  |  | 2.30 (-0.21; 4.91) | 0.07 |

BMI, body mass index; CVD, cardiovascular disease; HRT, hormone replacement therapy. Transferrin levels were corrected for CRP as suggested by BRINDA protocol prior to analysis. Statistical analysis by linear mixed-effect models.

**Table S7.** Longitudinal associations between log-transformed ferritin levels and CVD risk factors in the baseline, first, and second follow-ups of the CoLaus study, Lausanne, Switzerland.

|  | **Total** |  | **Total** |  | **Pre-menopause** |  | **Menopause** |  | **Perimenopausal** |  |
| --- | --- | --- | --- | --- | --- | --- | --- | --- | --- | --- |
|  | **Model 1** |  | **Model 2** |  | **Model 3** |  | **Model 3** |  | **Model 3** |  |
|  | **Beta (95% CI)** | **P-value** | **Beta (95% CI)** | **P-value** | **Beta (95% CI)** | **P-value** | **Beta (95% CI)** | **P-value** | **Beta (95% CI)** | **P-value** |
| **sample size** | **1482** |  | **1482** |  | **182** |  | **813** |  | **487** |  |
| **Glucose (mmol/L)** |  |  |  |  |  |  |  |  |  |  |
| T1 | Reference |  | Reference |  | Reference |  | Reference |  | Reference |  |
| T2 | 0.002 (-0.20; 0.20) | 0.97 | 0.02 (-0.12; 0.17) | 0.74 | -0.16 (-0.54; 0.06) | 0.08 | 0.25 (-0.15; 0.42) | 0.45 | -0.11 (-0.28; 0.07) | 0.20 |
| T3 | 0.11 (-0.05; 0.20) | 0.19 | 0.09 (-0.02; 0.22) | 0.13 | 0.10 (-0.06; 1.02) | 0.07 | 0.23 (0.02; 0.45) | 0.06 | -0.12 (-0.36; 0.07) | 0.24 |
| p for trend | 0.17 |  | 0.11 |  | 0.83 |  | 0.05 |  | 0.15 |  |
| **Insulin (mIU/mL)** |  |  |  |  |  |  |  |  |  |  |
| T1 | Reference |  | Reference |  | Reference |  | Reference |  | Reference |  |
| T2 | -0.01 (-0.14; 0.11) | 0.77 | 0.02 (-0.07; 0.09) | 0.63 | -0.09 (-0.32; 0.18) | 0.74 | -0.04 (-1.84; 1.98) | 0.65 | 0.10 (-0.05; 0.18) | 0.92 |
| T3 | 0.03 (-0.08; 0.14) | 0.58 | 0.06 (-0.02; 0.15) | 0.22 | 0.26 (-0.18; 0.90) | 0.34 | 0.05 (-1.91; 1.03) | 0.42 | 0.06 (-0.11; 0.21) | 0.79 |
| p for trend | 0.55 |  | 0.22 |  | 0.65 |  | 0.43 |  | 0.77 |  |
| **SBP (mmHg)** |  |  |  |  |  |  |  |  |  |  |
| T1 | Reference |  | Reference |  | Reference |  | Reference |  | Reference |  |
| T2 | 1.37 (-2.69; 5.44) | 0.97 | 3.76 (-0.24; 7.42) | 0.08 | 0.44 (-7.8; 8.01) | 0.10 | 3.42 (-5.92; 8.77) | 0.32 | 4.75 (-0.45; 8.00) | 0.38 |
| T3 | -0.26 (-3.74; 3.23) | 0.77 | 0.18 (-3.67; 3.10) | 0.90 | -10.2 (-25.8; 4.21) | 0.16 | 0.44 (-6.10; 5.20) | 0.87 | -2.24 (-8.71; 5.22) | 0.59 |
| p for trend | 0.77 |  | 0.22 |  | 0.06 |  | 0.86 |  | 0.73 |  |
| **DBP (mmHg)** |  |  |  |  |  |  |  |  |  |  |
| T1 | Reference |  | Reference |  | Reference |  | Reference |  | Reference |  |
| T2 | -0.28 (-2.78; 2.22) | 0.82 | -1.31 (-1.73; 2.04) | 0.28 | 0.29 (-6.30; 1.60) | 0.19 | -0.66 (-4.19; 3.75) | 0.74 | 3.84 (-.74; 5.36) | 0.92 |
| T3 | -0.66 (-2.80; 1.48) | 0.54 | -0.49 (-2.50; 1.48) | 0.63 | -10.1 (-22.3; 3.10) | 0.10 | -0.34 (-3.08; 2.02) | 0.80 | -0.35 (-4.45; 6.30) | 0.79 |
| p for trend | 0.54 |  | 0.84 |  | 0.07 |  | 0.90 |  | 0.33 |  |
| **HDL (mmol/L)** |  |  |  |  |  |  |  |  |  |  |
| T1 | Reference |  | Reference |  | Reference |  | Reference |  | Reference |  |
| T2 | -0.00 (-0.12; 0.11) | 0.97 | -0.29 (-0.11; 0.09) | 0.85 | 0.14 (-0.32; 0.33) | 0.94 | -0.01 (-0.23; 0.13) | 0.52 | -0.12 (-0.28; 0.13) | 0.20 |
| T3 | 0.01 (-0.08; 0.11) | 0.76 | -0.09 (-0.08; 0.10) | 0.80 | 0.17 (-0.40; 0.76) | 0.46 | 0.003(-0.14; 0.13) | 0.54 | -0.14 (-0.33; 0.06) | 0.12 |
| p for trend | 0.74 |  | 0.75 |  | 0.64 |  | 0.83 |  | 0.23 |  |
| **TC (mmol/L)** |  |  |  |  |  |  |  |  |  |  |
| T1 | Reference |  | Reference |  | Reference |  | Reference |  | Reference |  |
| T2 | 0.002 (-0.32; 0.14) | 0.97 | -0.18 (-0.32; 0.13) | 0.26 | -0.25 (-0.82; 0.27) | 0.12 | -0.22 (-0.42; 0.34) | 0.44 | -0.10 (-0.46; 0.21) | 0.52 |
| T3 | 0.09 (-0.13; 0.26) | 0.13 | 0.07 (-0.12; 0.26) | 0.52 | 0.41 (-0.53; 1.66) | 0.48 | 0.09 (0.18; 0.45) | 0.36 | -0.13 (-0.38; 0.45) | 0.84 |
| p for trend | 0.11 |  | 0.38 |  | 0.70 |  | 0.64 |  | 0.62 |  |

HDL-C, High-density lipoprotein cholesterol; BMI, body mass index; SBP, systolic blood pressure; DBP, diastolic blood pressure; TC, Total cholesterol; TSAT, transferrin saturation; CVRs, cardiovascular risk factors. All iron biomarkers and insulin were log-transformed. Model 1 included age. Model 2 included age, BMI, smoking, alcohol use, educational levels, hormone replacement therapy (HRT), antidiabetic and antihypertensive drugs, CVD, and menopause status, when the outcome was insulin or glucose, diabetes was also included in the model. Model 3 stratified by menopause status and included age, BMI, smoking, alcohol use, educational levels, hormone replacement therapy (HRT), antidiabetic and antihypertensive drugs, CVD, when the outcome was insulin or glucose, diabetes was also included in the model. All iron biomarkers were corrected for CRP as suggested by BRINDA protocol prior to analysis. Statistical analysis by multiple linear regression. Statistical analysis by linear mixed-effect models.

**Table S8.** Longitudinal associations between log-transformed transferrin levels and CVD risk factors in the baseline, first, and second follow-ups of the CoLaus study, Lausanne, Switzerland.

|  | **Total** |  | **Total** |  | **Pre-menopause** |  | **Menopause** |  | **Perimenopausal** |  |
| --- | --- | --- | --- | --- | --- | --- | --- | --- | --- | --- |
|  | **Model 1** |  | **Model 2** |  | **Model 3** |  | **Model 3** |  | **Model 3** |  |
|  | **Beta (95% CI)** | **P-value** | **Beta (95% CI)** | **P-value** | **Beta (95% CI)** | **P-value** | **Beta (95% CI)** | **P-value** | **Beta (95% CI)** | **P-value** |
| **sample size** | **1482** |  | **1482** |  | **182** |  | **813** |  | **487** |  |
| **outcome** |  |  |  |  |  |  |  |  |  |  |
| **Glucose (mmol/L)** |  |  |  |  |  |  |  |  |  |  |
| **T1** | Reference |  | Reference |  | Reference |  | Reference |  | Reference |  |
| **T2** | -0.01 (-0.20; 0.17) | 0.91 | -0.01 (-0.12; 0.15) | 0.95 | 0.14 (-0.13; 0.43) | 0.30 | -0.09 (-0.31; 0.15) | 0.50 | -0.006 (-0.21;0.19) | 0.50 |
| **T3** | -0.19 (-0.41; 0.18) | 0.07 | -0.11 (-0.27; 0.04) | 0.16 | 0.02 (-0.25; 0.30) | 0.58 | -0.35 (-0.63; -0.04) | 0.02 | 0.03 (-0.14; 0.22) | 0.91 |
| **p for trend** | 0.06 |  | 0.13 |  | 0.87 |  | 0.02 |  | 0.69 |  |
| **Insulin (mIU/mL)** |  |  |  |  |  |  |  |  |  |  |
| **T1** | Reference |  | Reference |  | Reference |  | Reference |  | Reference |  |
| **T2** | 0.003 (-0.11; 0.12) | 0.95 | 0.03 (-0.05; 0.13) | 0.42 | -0.14 (-0.42; 0.14) | 0.34 | 0.11 (-0.02; 0.25) | 0.06 | 0.03 (-0.13; 0.21) | 0.71 |
| **T3** | -0.08 (-0.22; 0.05) | 0.21 | 0.04 (-0.06; 0.14) | 0.46 | -0.12 (-0.40; 0.15) | 0.37 | 0.18 (0.00; 0.35) | 0.04 | -0.01 (-0.18; 0.13) | 0.82 |
| **p for trend** | 0.49 |  | 0.48 |  | 0.49 |  | 0.04 |  | 0.81 |  |
| **SBP (mmHg)** |  |  |  |  |  |  |  |  |  |  |
| **T1** | Reference |  | Reference |  | Reference |  | Reference |  | Reference |  |
| **T2** | -2.13 (-5.85; 1.58) | 0.35 | -1.50 (-5.39; 1.84) | 0.35 | 2.69 (-6.94; 12.33) | 0.68 | -4.47 (-10.33;1.35) | 0.13 | 1.69 (-4.43; 7.26) | 0.91 |
| **T3** | -3.14 (-7.38; 1.09) | 0.54 | -1.46 (-5.58; 2.69) | 0.54 | 2.39 (-6.89; 11.93) | 0.62 | -3.42 (-10.89;3.72) | 0.35 | 0.23 (-5.33; 5.21) | 0.95 |
| **p for trend** | 0.16 |  | 0.61 |  | 0.97 |  | 0.40 |  | 0.94 |  |
| **DBP (mmHg)** |  |  |  |  |  |  |  |  |  |  |
| **T1** | Reference |  | Reference |  | Reference |  | Reference |  | Reference |  |
| **T2** | -1.12 (-3.43; 1.15) | 0.33 | -0.47 (-2.75; 1.61) | 0.88 | 3.24 (-4.14; 10.72) | 0.41 | -1.80 (-4.93; 1.38) | 0.26 | 1.50 (-2.44; 5.44) | 0.45 |
| **T3** | -3.02 (-5.65; -0.42) | 0.02 | -1.38 (-3.87; 1.11) | 0.75 | 3.32 (-3.93; 10.61) | 0.37 | -3.71 (-7.55; 0.34) | 0.06 | -0.93 (-4.49; 2.61) | 0.60 |
| **p for trend** | 0.02 |  | 0.42 |  | 0.29 |  | 0.06 |  | 0.40 |  |
| **HDL (mmol/L)** |  |  |  |  |  |  |  |  |  |  |
| **T1** | Reference |  | Reference |  | Reference |  | Reference |  | Reference |  |
| **T2** | 0.15 (0.04; 0.26) | <0.001 | 0.10 (0.02; 0.21) | 0.01 | 0.10 (-0.34; 0.26) | 0.14 | 0.10 (-0.03; 0.25) | 0.12 | 0.13 (-0.05; 0.31) | 0.15 |
| **T3** | 0.10 (-0.02; 0.22) | 0.10 | 0.03 (-0.08; 0.14) | 0.57 | 0.01 (-0.31; 0.27) | 0.92 | -0.01 (-0.17; 0.18) | 0.89 | 0.006 (-0.1; 0.17) | 0.93 |
| **p for trend** | 0.83 |  | 0.68 |  | 0.94 |  | 0.49 |  | 0.96 |  |
| **TC (mmol/L)** |  |  |  |  |  |  |  |  |  |  |
| **T1** | Reference |  | Reference |  | Reference |  | Reference |  | Reference |  |
| **T2** | 0.15 (-0.06; 0.36) | 0.16 | 0.17 (-0.04; 0.38) | 0.10 | 0.24 (-0.38; 0.88) | 0.44 | 0.006 (-0.30; 0.31) | 0.96 | 0.26 (-0.11; 0.64) | 0.83 |
| **T3** | 0.09 (-0.15; 0.33) | 0.45 | 0.12 (-0.12; 0.36) | 0.33 | 0.30 (-0.31; 0.92) | 0.34 | 0.02 (-0.36; 0.41) | 0.89 | 0.01 (-0.35; 0.36) | 0.19 |
| **p for trend** | 0.38 |  | 0.40 |  | 0.37 |  | 0.87 |  | 0.92 |  |

HDL-C, High-density lipoprotein cholesterol; BMI, body mass index; SBP, systolic blood pressure; DBP, diastolic blood pressure; TC, Total cholesterol; TSAT, transferrin saturation; CVRs, cardiovascular risk factors. All iron biomarkers and insulin were log-transformed. Model 1 included age. Model 2 included age, BMI, smoking, alcohol use, educational levels, hormone replacement therapy (HRT), antidiabetic and antihypertensive drugs, CVD, and menopause status, when the outcome was insulin or glucose, diabetes was also included in the model. Model 3 stratified by menopause status and included age, BMI, smoking, alcohol use, educational levels, hormone replacement therapy (HRT), antidiabetic and antihypertensive drugs, CVD, when the outcome was insulin or glucose, diabetes was also included in the model. All iron biomarkers were corrected for CRP as suggested by BRINDA protocol prior to analysis. Statistical analysis by multiple linear regression. Statistical analysis by linear mixed-effect models.

**Table S9.** Longitudinal associations between log-transformed transferrin saturation levels and CVD risk factors in the baseline, first, and second follow-ups of the CoLaus study, Lausanne, Switzerland.

|  | **Total** |  | **Total** |  | **Pre-menopause** |  | **Menopause** |  | **Perimenopausal** |  |
| --- | --- | --- | --- | --- | --- | --- | --- | --- | --- | --- |
|  | **Model 1** |  | **Model 2** |  | **Model3** |  | **Model 3** |  | **Model 3** |  |
|  | **Beta (95% CI)** | **P-value** | **Beta (95% CI)** | **P-value** | **Beta (95% CI)** | **P-value** | **Beta (95% CI)** | **P-value** | **Beta (95% CI)** | **P-value** |
| **sample size** | **1482** |  | **1482** |  | **182** |  | **813** |  | **487** |  |
| **outcome** |  |  |  |  |  |  |  |  |  |  |
| **Glucose (mmol/L)** |  |  |  |  |  |  |  |  |  |  |
| **T1** | Reference |  | Reference |  | Reference |  | Reference |  | Reference |  |
| **T2** | 0.03 (-0.18; 0.25) | 0.93 | 0.06 (-0.10; 0.16) | 0.97 | -0.01 (-0.28; 0.25) | 0.89 | 0.08 (-0.25; 0.32) | 0.40 | 0.04 (-0.18; 0.19) | 0.50 |
| **T3** | -0.01 (-0.19; 0.16) | 0.78 | -0.007 (-0.12;0.21) | 0.18 | -0.009 (-0.24;0.21) | 0.92 | -0.02 (-0.22; 0.17) | 0.78 | -0.05 (-0.27; 0.12) | 0.47 |
| **p for trend** | 0.78 |  | 0.75 |  | 0.93 |  | 0.63 |  | 0.47 |  |
| **Insulin (mIU/mL)** |  |  |  |  |  |  |  |  |  |  |
| **T1** | Reference |  | Reference |  | Reference |  | Reference |  | Reference |  |
| **T2** | -0.06 (-0.20; 0.06) | 0.32 | -0.57 (-0.18; 0.03) | 0.42 | -0.04 (-0.33; 0.19) | 0.34 | -0.11 (-0.30; 0.05) | 0.16 | 0.03 (-0.21; 0.10) | 0.71 |
| **T3** | 0.03 (-0.07; 0.14) | 0.53 | 0.33 (-0.05; 0.12) | 0.46 | 0.15 (-0.07; 0.38) | 0.17 | -0.03 (-0.16; 0.09) | 0.54 | 0.10 (-0.07; 0.26) | 0.82 |
| **p for trend** | 0.39 |  | 0.31 |  | 0.18 |  | 0.88 |  | 0.33 |  |
| **SBP (mmHg)** |  |  |  |  |  |  |  |  |  |  |
| **T1** | Reference |  | Reference |  | Reference |  | Reference |  | Reference |  |
| **T2** | 1.83 (-2.46; 6.13) | 0.35 | 2.45 (-2.74; 5.64) | 0.35 | 2.08 (-6.89; 11.35) | 0.68 | 3.92 (-5.34; 9.32) | 0.13 | 2.25 (-3.93; 7.03) | 0.91 |
| **T3** | 1.98 (-1.52; 5.49) | 0.54 | 1.98 (-1.94; 4.90) | 0.54 | 0.18 (-7.58; 7.85) | 0.90 | 1.34 (-4.53; 6.25) | 0.35 | 3.77 (-2.22; 9.25) | 0.95 |
| **p for trend** | 0.31 |  | 0.61 |  | 0.46 |  | 0.40 |  | 0.44 |  |
| **DBP (mmHg)** |  |  |  |  |  |  |  |  |  |  |
| **T1** | Reference |  | Reference |  | Reference |  | Reference |  | Reference |  |
| **T2** | 1.09 (-1.55; 3.74) | 0.33 | 1.42 (-1.50; 3.55) | 0.88 | 2.82 (-4.47; 9.52) | 0.48 | 2.22 (-2.39; 5.54) | 0.26 | 0.88 (-3.22; 4.19) | 0.45 |
| **T3** | 1.25 (-0.90; 3.41) | 0.24 | 1.33 (-0.92; 3.20) | 0.08 | -0.93 (-6.85; 4.99) | 0.74 | 2.38 (-0.87; 4.96) | 0.06 | 2.41 (-1.56; 6.21) | 0.60 |
| **p for trend** | 0.22 |  | 0.32 |  | 0.74 |  | 0.06 |  | 0.40 |  |
| **HDL (mmol/L)** |  |  |  |  |  |  |  |  |  |  |
| **T1** | Reference |  | Reference |  | Reference |  | Reference |  | Reference |  |
| **T2** | 0.03 (-0.09; 0.15) | 0.59 | 0.04 (-0.07; 0.15) | 0.51 | 0.19 (-0.08; 0.47) | 0.14 | 0.13 (-0.04; 0.31) | 0.12 | -0.07 (-0.23; 0.09) | 0.40 |
| **T3** | 0.01 (-0.09; 0.11) | 0.50 | 0.08 (-0.08; 0.09) | 0.81 | -0.12 (-0.36; 0.11) | 0.92 | 0.09 (-0.04; 0.22) | 0.89 | -0.14 (-0.32; 0.02) | 0.10 |
| **p for trend** | 0.83 |  | 0.99 |  | 0.28 |  | 0.49 |  | 0.10 |  |
| **TC (mmol/L)** |  |  |  |  |  |  |  |  |  |  |
| **T1** | Reference |  | Reference |  | Reference |  | Reference |  | Reference |  |
| **T2** | -0.07 (-0.32; 0.17) | 0.56 | -0.05 (-0.29; 0.19) | 0.67 | 0.09 (-0.50; 0.70) | 0.44 | -0.016 (-0.44;0.32) | 0.75 | -0.09 (-0.41; 0.29) | 0.83 |
| **T3** | 0.03 (-0.17; 0.23) | 0.75 | 0.03 (-0.16; 0.23) | 0.70 | 0.22 (-0.29; 0.73) | 0.34 | 0.02 (-0.26; 0.03) | 0.84 | -0.11 (-0.49; 0.25) | 0.19 |
| **p for trend** | 0.65 |  | 0.61 |  | 0. 16 |  | 0.77 |  | 0.52 |  |

HDL-C, High-density lipoprotein cholesterol; BMI, body mass index; SBP, systolic blood pressure; DBP, diastolic blood pressure; TC, Total cholesterol; TSAT, transferrin saturation; CVRs, cardiovascular risk factors. All iron biomarkers and insulin were log-transformed. Model 1 included age. Model 2 included age, BMI, smoking, alcohol use, educational levels, hormone replacement therapy (HRT), antidiabetic and antihypertensive drugs, CVD, and menopause status, when the outcome was insulin or glucose, diabetes was also included in the model. Model 3 stratified by menopause status and included age, BMI, smoking, alcohol use, educational levels, hormone replacement therapy (HRT), antidiabetic and antihypertensive drugs, CVD , when the outcome was insulin or glucose, diabetes was also included in the model. All iron biomarkers were corrected for CRP as suggested by BRINDA protocol prior to analysis. Statistical analysis by multiple linear regression. Statistical analysis by linear mixed-effect models.
